# Clioquinol inhibits angiogenesis by promoting VEGFR2 degradation and synergizes with AKT inhibition to suppress triple-negative breast cancer vascularization

**DOI:** 10.1007/s10456-024-09965-1

**Published:** 2025-02-03

**Authors:** Yuan Gu, Tianci Tang, Moqin Qiu, Hongmei Wang, Emmanuel Ampofo, Michael D. Menger, Matthias W. Laschke

**Affiliations:** 1 Institute for Clinical and Experimental Surgery, Saarland University, 66421 Homburg, Saarland Germany; 2 Department of Respiratory Oncology, Guangxi Medical University Cancer Hospital, Nanning, 530021 China; 3 Shaanxi University of Chinese Medicine, Shaanxi, 712046 China

**Keywords:** Clioquinol, Angiogenesis, Endothelial cells, AKT inhibitor, Triple-negative breast cancer, VEGFR2 degradation

## Abstract

Inhibition of angiogenesis, either as monotherapy or in conjunction with other treatments, holds significant promise in cancer treatment. However, the limited efficacy of clinically approved anti-angiogenic agents underscores the urgent need for the development of novel drugs and therapeutic strategies. In this study, we demonstrate the highly selective inhibitory effects of clioquinol, a topical antifungal and antibiotic agent, on the angiogenic activity of endothelial cells (ECs) in a series of in vitro angiogenesis assays. Moreover, clioquinol effectively suppressed blood vessel formation in ex vivo aortic ring and in vivo Matrigel plug assays. Mechanistic studies revealed that clioquinol directly binds to the ATP-binding site of vascular endothelial growth factor receptor 2 (VEGFR2), promoting its degradation through both proteasome and lysosome pathways. This led to the down-regulation of the downstream extracellular signal-regulated kinase (ERK) pathway. In addition, the combination with the AKT inhibitor MK-2206 synergistically boosted the anti-angiogenic efficacy of clioquinol in vitro and in an in vivo dorsal skinfold chamber model of triple-negative breast cancer (TNBC), leading to the suppression of TNBC growth. Accordingly, clioquinol, either alone or in combination with AKT inhibitors, represents a promising therapeutic agent for future anti-angiogenic cancer treatment.

**Supplementary Information:**

The online version contains supplementary material available at 10.1007/s10456-024-09965-1.

## Introduction

Tumor angiogenesis refers to the development of a new microvascular network within a tumor, originating from preexisting blood vessels of the surrounding host tissue. It typically occurs when a tumor reaches a volume of 1–2 mm^3^ and can no longer be adequately supplied with oxygen and nutrients via diffusion [1]. During this process, endothelial cells (ECs) are activated by pro-angiogenic factors released from the tumor microenvironment (TME), such as vascular endothelial growth factor (VEGF) and basic fibroblast growth factor (FGF2) [2]. As a consequence, they proliferate and migrate towards the tumor tissue, where they ultimately establish a new microvascular network [3]. These newly formed blood vessels play a pivotal role in supporting tumor growth and metastasis [4], making them promising targets for anti-cancer therapy.

So far, approximately 20 anti-angiogenic agents, including neutralizing antibodies against VEGF and its receptors (VEGFRs), recombinant fusion proteins targeting VEGF, tyrosine kinase inhibitors, and mammalian target of rapamycin inhibitors, have been approved by the United States Food and Drug Administration (FDA) [5, 6]. Nevertheless, the clinical efficacy of these therapies is considerably limited by the emergence of tumor resistance and the occurrence of side effects [5, 7–9]. Thus, novel anti-angiogenic drugs and strategies that are efficient and safe are still urgently required.

One promising avenue to address this need is drug repurposing. Clioquinol is an antimicrobial agent, which is used for the topical treatment of skin infections. Originally, it was extensively applied as oral antibiotic for the therapy of diarrhea and indigestion until it became associated with an outbreak of subacute myelooptic neuropathy (SMON) in Japan [10]. In recent decades, clioquinol has regained attention as a potential therapeutic agent for neurodegenerative diseases as well as cancer [11–14]. In fact, previous studies demonstrated potent inhibitory effects of clioquinol on the growth of different cancer types, including B-cell lymphoma, ovarian cancer, ZIP1-deficient prostate tumor, and leukemia [15–17]. However, the molecular and cellular mechanisms contributing to the anti-cancer activity of clioquinol, particularly its anti-angiogenic activity, have not yet been fully elucidated.

Inhibition of AKT, a key downstream node of diverse receptor tyrosine kinases, G-protein-coupled receptors, and cytokine receptors, shows great promise for suppressing angiogenesis, halting tumor growth and overcoming drug resistance [18]. So far, several AKT inhibitors have been developed and evaluated in clinical trials. Among them, MK-2206 is a highly selective allosteric AKT inhibitor. Preclinical investigations in a broad spectrum of cancer types demonstrated the anti-cancer properties of MK-2206, particularly its capacity to enhance the effectiveness of various therapeutic approaches, such as chemotherapy, targeted therapies, and hormone therapy [19–22]. Phase II trials further reported that MK-2206 restores erlotinib activity in patients with advanced non-small cell lung cancer (NSCLC) and improves the response to paclitaxel and trastuzumab in patients with hormone receptor (HR)-negative and human epidermal growth factor receptor 2 (HER2)-positive breast cancer [23, 24]. Additionally, recent clinical trials have shown that the combination of the ATP-competitive AKT inhibitor capivasertib with docetaxel enhances the outcomes for patients with advanced prostate cancer [25], while its combination with fulvestrant significantly improves progression-free survival among patients with HR-positive advanced breast cancer [26]. These findings strongly suggest that combining AKT inhibitors with other treatments represents a promising approach to overcome therapeutic resistance and improve clinical outcomes.

Based on these findings, we first compared in the present study the sensitivity of breast cancer cells, ECs, pericytes, and fibroblasts to clioquinol treatment. We then narrowed our focus to ECs, examining the impact of clioquinol on their angiogenic activity in vitro and in vivo. Furthermore, we elucidated the precise molecular mechanisms underlying the anti-angiogenic activity of clioquinol. In addition, we evaluated the effects of clioquinol alone and in combination with MK-2206 on the vascularization and growth of triple-negative breast cancer (TNBC) in a mouse dorsal skinfold chamber model.

## Materials and methods

### Study design

In this study, sample size was determined based on previous publications. For in vitro assays, at least 3 independent experiments were conducted, each comprising a minimum of 3 biological replicates (i.e. independent cell cultures). For mouse experiments, each group included at least 5 animals. Randomization was performed for group allocation in the dorsal skinfold chamber model. Data analysis was conducted by investigators blinded to group assignments. No samples or animals were excluded. Detailed n values for each assay are provided in the figure legends.

### Chemicals

Clioquinol, cycloheximide, and MG132 were purchased from Santa Cruz Biotechnology (Heidelberg, Germany). Chloroquine diphosphate salt was purchased from Sigma-Aldrich (Taufkirchen, Germany). Lenvatinib, tivozanib, and MK-2206 2HCl (MK-2206) were purchased from MedChemExpress (NJ, USA). Dimethyl sulfoxide (DMSO) was purchased from PanReac Applichem (Darmstadt, Germany).

### Cell culture

Human umbilical vein endothelial cells (HUVECs), human dermal microvascular endothelial cells (HDMECs), and human pericytes from placenta (hPC-PLs) were purchased from PromoCell (Heidelberg, Germany). HUVECs were cultured in Endothelial Cell Growth Medium (EGM; PromoCell), HDMECs in EGM-MV (PromoCell), and hPC-PLs in Growth Medium 2 (PromoCell), all supplemented with the corresponding SupplementMix from PromoCell. Normal human dermal fibroblasts (NHDFs), generously provided by Dr. Wolfgang Metzger (Saarland University), were cultured in Dulbecco’s modified Eagle’s medium (PAA, Cölbe, Germany) supplemented with 10% fetal calf serum (FCS), 100 U/mL penicillin (PAA), and 0.1 mg/mL streptomycin (PAA). The murine luciferase-expressing TNBC cell line 4T1-Luc2 (RRID: CVCL_A4BM), the human TNBC cell line MDA-MB-231 (RRID: CVCL_0062), and the human non-TNBC cell line MCF-7 (RRID: CVCL_0031) were purchased from ATCC (Wesel, Germany) and cultured in RPMI 1640 medium with 10% FCS, 100 U/mL penicillin, and 0.1 mg/mL streptomycin. All cells were maintained in a humidified incubator at 37 °C with 5% CO_2_.

### Water-soluble tetrazolium (WST)-1 assay

The viability of cells was evaluated by means of WST-1 assays. For this purpose, cells seeded in 96-well plates were exposed to various treatments. After 48 h, 10 µL WST-1 reagent (Roche Diagnostics, Mannheim, Germany) was added to each well and incubated at 37 °C for 30 min. Absorbance was measured at 450 nm with 620 nm as reference using a PHOmo microplate reader (anthos Mikrosysteme GmbH, Krefeld, Germany). Cell viability was expressed as percentage relative to the control group.

### Lactate dehydrogenase (LDH) assay

The cytotoxicity of compounds was assessed using LDH assays. For this purpose, HUVECs seeded in 96-well plates were exposed to various concentrations of compounds. After 24 h of treatment, 100 µL LDH reaction mix (Roche Diagnostics) was added to each well and incubated at room temperature for 10 min, followed by addition of 50 µL stop solution (Roche Diagnostics). Absorbance was measured at 492 nm with 620 nm as reference using a microplate photometer (PHOmo). The cytotoxicity of each compound was expressed as percentage relative to the high control group, in which all cells were lysed.

### Flow cytometry

The proliferating activity of ECs was evaluated by bromodeoxyuridine (BrdU) incorporation assays following established protocols [27]. HUVECs were exposed to various concentrations of clioquinol. After 6 h of treatment, BrdU was added into each well at a final concentration of 10 µM. After culture for another 18 h, the cells were fixed with 70% ethanol and then incubated with a fluorescein isothiocyanate-labeled anti-BrdU antibody (Cat# 11-5071-42; RRID: AB_11042627; Thermo Fisher Scientific, Karlsruhe, Germany) for 30 min. Cell samples were then analyzed using a FACSLyric flow cytometer (BD Biosciences, Heidelberg, Germany) to quantify BrdU-positive cells. 10,000 events were acquired for each sample and the analysis was performed using FACSuite™ Software (BD Biosciences). EC proliferation was expressed as percentage relative to the control group.

To investigate the effects of clioquinol on the expression of membrane VEGFR2, HUVECs were treated with 0.1% DMSO or 10 µM clioquinol for 0.5, 1, 2, 3, and 4 h. The cells were then harvested using Accutase (PAN-Biotech GmbH, Aidenbach, Germany) and incubated with an anti-VEGFR2 antibody conjugated to PE (Cat# 130-120-480; RRID: AB_2801769; Miltenyi Biotec, Bergisch Gladbach, Germany) for 30 min at room temperature, followed by analysis using a FACSLyric flow cytometer (BD Biosciences). Membrane VEGFR2 expression was expressed as percentage relative to the control group at 0.5 h.

### Transwell migration assay

The migratory activity of ECs was assessed via transwell migration assays. For this purpose, HUVECs were exposed to various concentrations of clioquinol for 24 h. Following treatment, 1.5 × 10^5^ treated cells were suspended in 500 µL Endothelial Basal Medium (EBM; PromoCell) and seeded into each polycarbonate membrane insert in a 24-well transwell plate (8 μm pores; Corning; Merck KGaA, Darmstadt, Germany), with 750 µL EBM containing 1% FCS added to each well. After 5 h of incubation, unmigrated cells were removed and migrated cells were stained with Diff-Quick (LT-SYS Diagnostika, Berlin, Germany). EC migration was quantified by counting the number of migrated cells in 20 regions of interest using a BZ-8000 microscope (Keyence, Osaka, Japan) and expressed as percentage relative to the control group.

### Tube formation assay

The tube-forming activity of ECs was analyzed by tube formation assays. In a first step, 1.5 × 10^4^ HUVECs were suspended in EGM containing different compounds and seeded into Matrigel-coated wells of a 96-well plate. After 24 h of incubation, newly formed tubes were imaged using a phase-contrast microscope (BZ-8000; Keyence). EC tube formation was quantified by analyzing the number of tube meshes per well using ImageJ software with the angiogenesis analyzer plug-in (U.S. National Institutes of Health, Bethesda, Maryland, USA) and expressed as percentage relative to the control group.

### Spheroid sprouting assay

As outlined previously [28], HUVECs were seeded in non-adherent round bottom 96-well plates (500 cells per well) with EGM containing 0.24% (w/v) methylcellulose. After culture for 24 h, formed spheroids were harvested and suspended in EBM containing 10% FCS, 0.25% (w/v) methylcellulose, and 1 mg/mL rat collagen (Serva, Heidelberg, Germany). About 50 spheroids were then transferred to each well of pre-warmed 24-well plates. Following a 45-minute incubation, spheroids were exposed to different treatments for 24 h and imaged using a phase-contrast microscope (DFC450C; Leica Microsystems, Wetzlar, Germany). Spheroid sprouting was quantified by measuring the cumulative length of sprouts using LAS V4.8 software (Leica Microsystems) and expressed as percentage relative to the control group.

### Aortic ring assay

The thoracic aorta from a BALB/c mouse (RRID: IMSR_RJ: BALB-CANNRJ; Janvier-Labs, Le Genest, France). was cut into 0.5-mm rings and embedded in Matrigel (Corning; Merck KGaA) in 96-well plates (one ring per well). After a 15-minute incubation, the rings were exposed to different concentrations of clioquinol. Following treatment for 6 days, the aortic rings were imaged using a phase-contrast microscope (BZ-8000; Keyence). Aortic sprouting was quantified by measuring the sprouting area and expressed as percentage relative to the control group.

### Western blotting

As previously detailed [29], the treated cells were lysed on ice for 10 min in RIPA lysis buffer (Thermo Fisher Scientific) supplemented with Protease Inhibitor Cocktail (Sigma-Aldrich), and then collected using a cell scraper. Following centrifugation, the supernatant from the cell lysate was collected for protein quantification using the Pierce BCA Protein Assay Kit (Thermo Fisher Scientific). Protein samples (10 µg) were then separated via sodium dodecyl sulphate-polyacrylamide gel electrophoresis and then transferred onto polyvinylidene difluoride membranes (Bio-Rad Laboratories, Munich, Germany). Proteins of interest were detected using specific antibodies, including rabbit monoclonal anti-phosphorylated (p)-VEGFR2 antibody (1:250; Cat# 2478; RRID: AB_331377; Cell Signaling Technology, Frankfurt, Germany), rabbit monoclonal anti-VEGFR2 antibody (1:250; Cat# 9698; RRID: AB_11178792; Cell Signaling Technology), rabbit polyclonal anti-VEGFR1 antibody (1:250; Cat# ab32152; RRID: AB_778798; Abcam, Cambridge, UK), rabbit monoclonal anti-FGF receptor 1 (FGFR1) antibody (1:250; Cat# 9740; RRID: AB_11178519; Cell Signaling Technology), rabbit monoclonal anti-Tie2 antibody (1:250; Cat# 7403; RRID: AB_10949315; Cell Signaling Technology), rabbit monoclonal anti-p-FAK antibody (1:250; Cat# 8556; RRID: AB_10891442; Cell Signaling Technology), rabbit polyclonal anti-FAK antibody (1:250; Cat# 3285; RRID: AB_2269034; Cell Signaling Technology), rabbit monoclonal anti-p-AKT antibody (1:500; Cat# 4060; RRID: AB_2315049; Cell Signaling Technology), rabbit monoclonal anti-AKT antibody (1:500; Cat# 4685; RRID: AB_2225340; Cell Signaling Technology), mouse monoclonal anti-p-ERK antibody (1:500; Cat# ab50011; RRID: AB_1603684; Abcam), rabbit polyclonal anti-ERK antibody (1:500; Cat# ab115799; RRID: AB_10902111; Abcam), and mouse monoclonal anti-β-actin antibody (1:3000; Cat# A5441; RRID: AB_476744; Sigma-Aldrich). This was followed by incubation with an anti-rabbit (1:1000; Cat# HAF008; RRID: AB_357235; R&D Systems, Wiesbaden, Germany) or anti-mouse (1:1000; Cat# HAF007; RRID: AB_357234; R&D Systems) horseradish peroxidase-conjugated secondary antibody. Protein signals were visualized using an enhanced chemiluminescence kit (GE Healthcare, Freiburg, Germany) and imaged with a ChemoCam Imager (Intas, Göttingen, Germany). The protein expression level was quantified using ImageJ software, normalized to β-actin or its unphosphorylated form and expressed as percentage relative to the control group.

### Quantitative real-time polymerase chain reaction (PCR)

Total RNA was extracted from treated HUVECs using the RNeasy Mini kit (Qiagen, Hilden, Germany) according to the manufacturer’s protocol. Subsequently, 1 µg RNA was reverse transcribed using the QuantiNova Reverse Transcription Kit (Qiagen). Quantitative real-time PCR was conducted on a MiniOpticon Real-Time PCR System (Bio-Rad Laboratories) using the QuantiNova SYBR green PCR kit (Qiagen). The messenger RNA (mRNA) level of VEGFR2 was determined using the 2^−ΔΔCt^ method with GAPDH as endogenous control and expressed as percentage relative to the control group. The primer sequences were as follows: 5′-GGCCCAATAATCAGAGTGGCA-3′ (forward) and 5′-CCAGTGTCATTTCCGATCACTTT-3′ (reverse) for human VEGFR2; 5′-ATGGGTGTGAACCATGAGAAGTA-3′ (forward) and 5′-GGCAGTGATGGCATGGAC-3′ (reverse) for human GAPDH.

### Molecular docking

To generate the three-dimensional (3D) binding modes of compounds within the VEGFR2 kinase domain, we employed the PyMOL Molecular Graphics System (Version 1.5.0.4, Schrödinger, LLC, New York, NY, USA), Coot, and Materials Studio 6.0 software (Accelrys Inc, USA). These tools facilitated the exploration of intermolecular interactions, including hydrogen bonding, van der Waals forces, electrostatic interactions, and hydrophobic interactions, in solving the Newtonian equations of motion for atoms in protein and compound molecules iteratively. Consequently, the molecular conformation underwent changes throughout the simulation. In line with thermodynamic principles, the system strived to attain its lowest free energy state, representing the utmost stability. Subsequently, the simulation was utilized to analyze the crystal structures of VEGFR2, obtained from the Protein Data Bank (RCSB PDB; http://www.rcsb.org) under accession codes 3WZD (VEGFR2-lenvatinib complex) and 4ASE (VEGFR2-tivozanib complex).

### Measurement of intracellular ATP

Intracellular levels of ATP were measured using the Firefly Luciferase ATP Assay Kit (Cell Signaling Technology) following the manufacturer’s protocol with minor modifications. Briefly, HUVECs seeded in a 96-well plate were treated with or without 1 mM ATP. After 2 h, the cells were rinsed with PBS for 3 times and lysed in 100 µL Cell Lysis Buffer. Then, 100 µL Firefly Luciferase Reaction Mixture was added to each well. After shaking the plate for 2 min, chemiluminescence was measured in each well using a Tecan Infinite M200 PRO luminometer (Crailsheim, Germany). The intracellular ATP level was expressed as percentage relative to the control group.

### Cell-free VEGFR2 kinase assay

The interaction between clioquinol and VEGFR2 was evaluated in vitro using the ADP-Glo™ Kinase Assay (Promega, Walldorf, Germany) and the VEGFR2 Kinase Enzyme System (Promega). All assays were performed in white 96-well flat-bottom plates. Each reaction mixture (25 µL) contained 10 ng recombinant VEGFR2 (amino acids 789 to end), 0.2 µg/µL Poly (4:1 Glu, Tyr) Peptide Substrate, serial dilutions of clioquinol, and ATP at a final concentration of 10 µM. For ATP competition experiments, the reaction mixture (25 µL) contained 0.2 µg/µL Peptide Substrate, different concentrations of lenvatinib or clioquinol, 10 or 500 µM ATP, 10 or 40 ng recombinant VEGFR2, respectively. Reactions were incubated at room temperature for 60 min. Then, 25 µL ADP-Glo™ Reagent was added to each well to terminate the kinase reaction and deplete remaining ATP, followed by a 40-minute incubation at room temperature. Subsequently, 50 µL Kinase Detection Reagent was added to each well to convert ADP to ATP. After another 40-minute incubation at room temperature, the generated ATP correlating with the kinase activity was measured using a luciferase/luciferin reaction with a Tecan Infinite M200 PRO luminometer. VEGFR2 kinase activity was expressed as percentage relative to the control group. The IC_50_ value of clioquinol at 10 µM ATP was calculated using GraphPad Prism 9 software.

### Mouse experiments

The Matrigel plug assay was performed following the protocol outlined in a previous study [30]. A mixture of Matrigel with murine VEGF (1 µg/mL; R&D Systems), murine FGF2 (1 µg/mL; R&D Systems), heparin (60 IU/mL; B. Braun, Melsungen, Germany), and either 0.1% DMSO or 10 µM clioquinol was injected subcutaneously into the flanks of 8-week-old BALB/c mice (7 mice per group; Janvier-Labs). The mice were anesthetized with isoflurane (5% induction and 2% maintenance) during the injection. Following a 7-day period, the Matrigel plugs were collected for subsequent immunohistochemical analyses.

The dorsal skinfold chamber model was conducted as described previously in detail [30, 31]. Tumor spheroids were generated by culturing 4T1-Luc2 cells in agarose-coated 96-well plates for 3 days. One day after cell seeding, the dorsal skinfold chambers were surgically implanted into female 3-4-month-old BALB/c mice (Janvier-Labs). The mice were anesthetized with an intraperitoneal injection of 90 mg/kg body weight ketamine (Serumwerke Bernburg AG, Bernburg, Germany) and 12 mg/kg body weight xylazine (Bayer, Leverkusen, Germany) before the operation, and were given a subcutaneous injection of 10 mg/kg body weight carprofen (Cp-Pharma, Burgdorf, Germany) for analgesia after the operation. Two days later, one tumor spheroid was transplanted into each chamber. The mice were then randomly allocated into 4 groups (10 mice per group) and treated with intraperitoneal injections of 30 mg/kg body weight clioquinol (dissolved in a 1:4 ratio of DMSO to corn oil) once daily, 80 mg/kg body weight MK-2206 (dissolved in 30% SBE-β-CD in NaCl; MedChemExpress) every two days, a combination of clioquinol and MK-2206 with doses mentioned above, or vehicle (control). The vehicle group received daily intraperitoneal injections of a 1:4 ratio of DMSO to corn oil (40 µL) and an intraperitoneal injection of 30% SBE-β-CD (40 µL) every two days. Tumor growth and vascularization were monitored on days 0, 3, 6, 10, and 14 after spheroid transplantation by means of stereomicroscopy and intravital fluorescence microscopy with recordings analyzed offline using CapImage (Zeintl, Heidelberg, Germany). The analyses included the quantification of tumor size, functional microvessel density, microvessel diameter, centerline red blood cell (RBC) velocity, and volumetric blood flow [32, 33]. Additionally, tumor growth in mice randomly selected from the control and combination group (5 mice per group) was analyzed by bioluminescence imaging (IVIS Spectrum imager; PerkinElmer, MA, USA) on days 10 and 14 after spheroid transplantation. This was achieved by administering an intraperitoneal injection of 150 mg/kg body weight D-luciferin (PerkinElmer) with imaging conducted 17 min post-injection. At the end of the experiment, the tumor tissues were excised for further histological and immunohistochemical analyses.

All mice were housed under a 12-hour light/dark cycle with a controlled room temperature (22–24 °C) and humidity (40–60%), and had free access to food and water. They were acclimated for at least 7 days before the experiments. The humane endpoint was established when body weight loss exceeded 20%.

### Histology and immunohistochemistry

Formalin-fixed paraffin-embedded Matrigel plugs and tumor tissues were serially sliced into 3-µm sections. For the analysis of tumor size, sections with the largest area for each tumor were selected, stained with hematoxylin and eosin (H&E), imaged using a BZ-8000 microscope (Keyence), and subjected to planimetric tumor area measurements by means of an image analysis software (Keyence).

Microvessels in Matrigel plugs and tumors were detected by sequential staining with a rabbit anti-mouse CD31 antibody (1:150; Cat# ab182981; RRID: AB_2920881; Abcam), a goat anti-rabbit Alexa Fluor 555-labeled secondary antibody (1:150; Cat# A27039; RRID: AB_2536100; Thermo Fisher Scientific), and Hoechst 33342 (2 µg/mL; Sigma-Aldrich), followed by the observation under a fluorescence microscope (Olympus BX60). The microvessel density was determined by counting the number of all CD31-positive microvessels divided by the corresponding tissue area.

Tumor cell proliferation and apoptosis were assessed by sequential staining with a monoclonal rabbit anti-mouse Ki67 antibody (1:500; Cat# 12202; RRID: AB_2620142; Cell Signaling Technology) or a polyclonal rabbit anti-mouse cleaved caspase-3 antibody (1:150; Cat# 9661; RRID: AB_2341188; Cell Signaling Technology), biotinylated goat anti-rabbit secondary antibody (1:150; Cat# ab64256; RRID: AB_2661852; Abcam), streptavidin‐peroxidase conjugate (Abcam), and 3‐amino‐9‐ethylcarbazole substrate (Abcam), followed by counterstaining with Mayer’s hemalum solution (Merck KGaA). Percentages of Ki67-positive and cleaved caspase‐3-positive tumor cells were determined using a BX60 microscope (Olympus).

Microvascular VEGFR2 expression was analyzed by sequential staining with a monoclonal rat anti-mouse CD31 antibody (1:100; ab56299; Abcam), a monoclonal rabbit anti-mouse VEGFR2 antibody (1:100; Cat# 2479; RRID: AB_2212507; Cell Signaling Technology), a goat anti-rat Alexa Fluor488-labeled secondary antibody (1:150; Cat# A11006; RRID: AB_2534074; Thermo Fisher Scientific), a goat anti-rabbit Cy3-conjugated secondary antibody (1:100; Cat# A10520; RRID: AB_10563288; Thermo Fisher Scientific), and Hoechst 33342 (2 µg/mL; Sigma-Aldrich). The analyses involved the quantification of the area of VEGFR2 signal normalized to CD31 area as well as the determination of the mean fluorescence intensity of VEGFR2 using ImageJ software.

### Calculation of the coefficient of drug interaction (CDI)

The combined effects of clioquinol and MK-2206 in the dorsal skinfold chamber model were assessed by calculating the value of CDI using the formula: CDI = AB/(A × B), where AB represents the ratio of the combination group to the control group, and A and B denote the ratios of each individual compound group to the control group. A CDI value of less than 1 indicates synergism, a value of 1 indicates additivity, and a value greater than 1 indicates antagonism.

### Statistics

The statistical analysis was performed utilizing GraphPad Prism 9 software. Differences between two groups were assessed using the unpaired two-tailed t-test, while differences among multiple groups were evaluated using One-Way ANOVA followed by Tukey’s multiple comparisons test. All data are expressed as means ± SEM. Statistical significance was defined as *P* < 0.05 (*P < 0.05; **P < 0.01; ***P < 0.001).

## Results

### Clioquinol selectively targets ECs

**Fig. 1:**
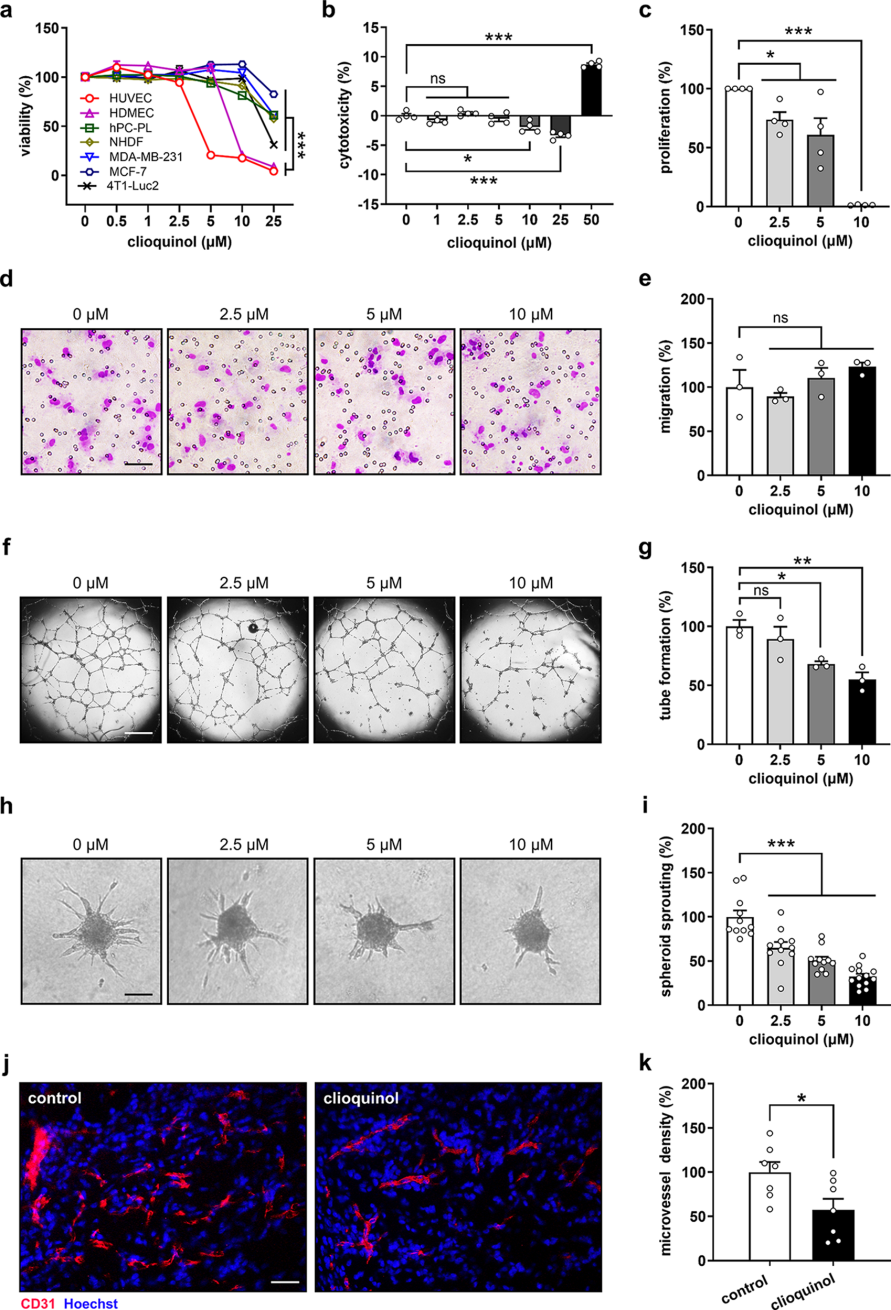
Clioquinol selectively targets ECs and inhibits angiogenesis. **a** Viability (% of 0 µM) of HUVECs, HDMECs, hPC-PLs, NHDFs, MDA-MB-231, MCF-7, and 4T1-Luc2 cells after 48-hour exposure to a serial dilution of clioquinol, as assessed by WST-1 assay (*n* = 4). **b** Cytotoxicity (% of total cell death) of clioquinol against HUVECs after 24-hour treatment, as assessed by LDH assay (*n* = 4). **c** Proliferation (% of 0 µM) of HUVECs treated with 0, 2.5, 5, or 10 µM clioquinol for 24 h, as assessed by BrdU incorporation assay (*n* = 4). **d** Light microscopic images of migrated HUVECs after 5-hour incubation. The cells were treated with 0, 2.5, 5, or 10 µM clioquinol for 24 h prior to this assay. Scale bar: 65 μm. **e** Migration (% of 0 µM) of HUVECs treated as described in (d) (*n* = 3). **f** Phase-contrast microscopic images of tube-forming HUVECs after 18-hour treatment with 0, 2.5, 5, or 10 µM clioquinol. Scale bar: 700 μm. **g** Tube formation (% of 0 µM) of HUVECs treated as described in (f) (*n* = 3). **h** Phase-contrast microscopic images of HUVEC spheroids after 24-hour treatment with 0, 2.5, 5, or 10 µM clioquinol. Scale bar: 95 μm. **i** Sprouting (% of 0 µM) of HUVEC spheroids treated as described in (h) (*n* = 11–13). **j** Fluorescence microscopic images of Matrigel plugs containing DMSO (control) or 10 µM clioquinol. The sections were stained with an anti-CD31 antibody (red) and Hoechst 33342 (blue) for the visualization of ECs and cell nuclei, respectively. Scale bar: 45 μm. **k** Microvessel density (% of control) of control and clioquinol-containing Matrigel plugs, as assessed by immunohistochemistry (*n* = 7). Means ± SEM. *P < 0.05, **P < 0.01, ***P < 0.001; ns, not significant. (a-c, e, g, i: one-way ANOVA with Tukey’s multiple comparisons test; k: unpaired Student’s t-test)

The anti-cancer potential of clioquinol has been demonstrated in several xenograft mouse models [15–17]. To elucidate the underlying cellular mechanisms, we compared the effects of this compound on different cell types, which are present in the TME of breast cancer and play crucial roles in tumor progression. These cells included breast cancer cells (MCF-7, MDA-MB-231, and 4T1-Luc2), ECs (HUVECs and HDMECs), pericytes (hPC-PLs), and fibroblasts (NHDFs). WST-1 assays revealed that treatment with 10 and 25 µM clioquinol for 48 h selectively and significantly reduced the viability of both tested EC types when compared to the other cell types (Fig. 1a).

### Clioquinol inhibits EC angiogenesis in vitro and in vivo

We next performed LDH assays to assess the cytotoxic effects of clioquinol on HUVECs after 24 h of treatment. Our results showed that clioquinol at concentrations up to 25 µM induces no cytotoxicity (Fig. 1b). Notably, clioquinol at 10 and 25 µM even decreased the cytotoxicity to negative values, indicating a reduction in LDH activity. Accordingly, we chose non-cytotoxic concentrations of 2.5, 5, and 10 µM for subsequent angiogenesis assays.

BrdU incorporation assays revealed that clioquinol inhibits HUVEC proliferation in a dose-dependent manner, with 10 µM of this compound completely blocking this process (Fig. 1c). Identical doses of clioquinol had no effect on HUVEC migration (Fig. 1d, e). However, treatment with 5 and 10 µM clioquinol significantly reduced EC tube formation by 32% and 45%, respectively (Fig. 1f, g). Moreover, we demonstrated that clioquinol effectively inhibits the sprouting of HUVEC spheroids (Fig. 1h, i). To confirm these in vitro findings, we additionally performed an ex vivo aortic ring assay. Our results showed that aortic rings exposed to clioquinol exhibit a dose-dependent reduction in their sprouting activity when compared to controls (Supplementary Fig. 1). In addition, we evaluated the effects of clioquinol on angiogenesis in an in vivo Matrigel plug assay. Matrigel plugs containing 10 µM clioquinol demonstrated a 43% reduction in microvessel density when compared to controls (Fig. 1j, k).

### Clioquinol down-regulates VEGFR2 in ECs

**Fig. 2:**
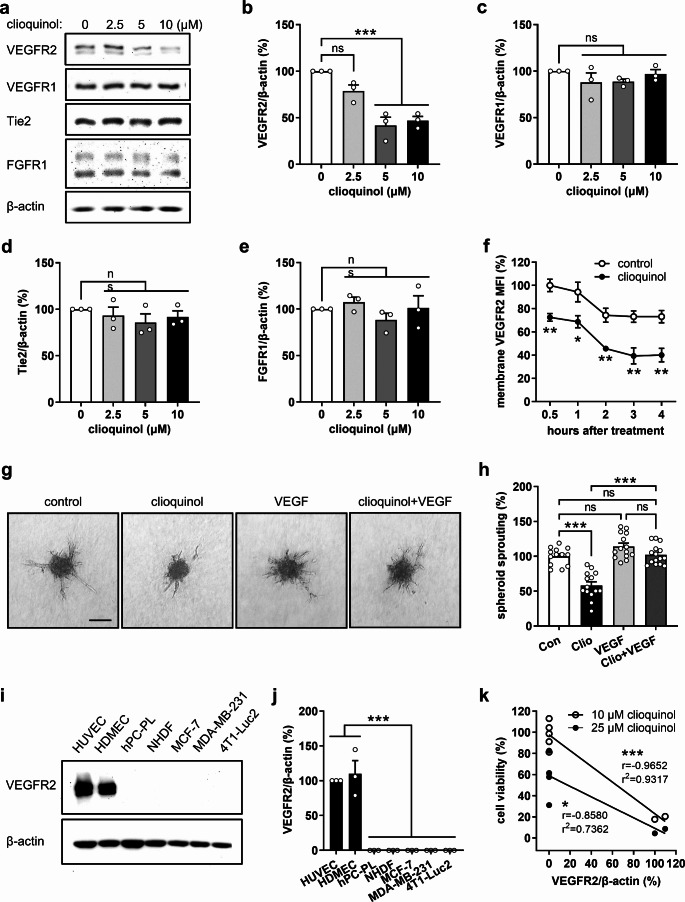
Clioquinol selectively down-regulates VEGFR2 in ECs. **a** Western blots showing VEGFR2, VEGFR1, Tie2, FGFR1, and β-actin expression in HUVECs after 4-hour treatment with 0, 2.5, 5, or 10 µM clioquinol. **b**-**e** Expression level (% of 0 µM) of VEGFR2 (**b**), VEGFR1 (**c**), Tie2 (**d**), and FGFR1 (**e**) normalized to β-actin in HUVECs treated as described in (a) (*n* = 3 independent experiments). **f** Mean fluorescence intensity (MFI) of membrane VEGFR2 on HUVECs treated with or without 10 µM clioquinol for 0.5, 1, 2, 3, and 4 h, as assessed by flow cytometry (*n* = 4). **g** Phase-contrast microscopic images of HUVEC spheroids after 24-hour treatment without or with clioquinol in the absence or presence of 25 ng/mL VEGF. Scale bar: 135 μm. **h** Sprouting (% of control) of HUVEC spheroids treated as described in (g) (*n* = 13–15). **i** Western blots showing VEGFR2 and β-actin expression in HUVECs, HDMECs, hPC-PLs, NHDFs, MCF-7, MDA-MB-231, and 4T1-Luc2 cells. **j** Expression level (% of HUVEC) of VEGFR2 normalized to β-actin in different cell types as described in (i) (*n* = 3 independent experiments). **k** Correlation between cell viability and VEGFR2 expression following exposure to 10 or 25 µM clioquinol. Means ± SEM. *P < 0.05, **P < 0.01, ***P < 0.001; ns, not significant. (b-e, h, j: one-way ANOVA with Tukey’s multiple comparisons test; f: unpaired Student’s t-test; k: Pearson correlation coefficient)

To uncover the molecular mechanisms underlying the anti-angiogenic effects of clioquinol, we examined the expression of several pivotal angiogenesis-related receptor tyrosine kinases, including VEGFR2, VEGFR1, Tie2, and FGFR1, in HUVECs treated with different concentrations of clioquinol for 4 h. Western blot analyses demonstrated that concentrations of 5 and 10 µM clioquinol selectively decrease the total levels of VEGFR2 in ECs, while leaving other tested receptors unaffected (Fig. 2a-e). Considering that VEGF typically binds to VEGFR2 on the EC membrane, which in turn activates downstream pro-angiogenic pathways, we then analyzed the effects of clioquinol on the expression of membrane VEGFR2. Flow cytometric analyses indicated that VEGFR2 on the EC surface underwent an internalization process over time (Fig. 2f). Of interest, this process was more pronounced in cells treated with 10 µM clioquinol when compared to controls (Fig. 2f).

To investigate whether VEGFR2 down-regulation contributes to the anti-angiogenic effect of clioquinol, HUVEC spheroids treated with DMSO or 10 µM clioquinol were exposed to the VEGFR2 ligand VEGF. Notably, the suppressive effects of clioquinol on EC spheroid sprouting were completely reversed by VEGF (Fig. 2g, h), indicating that clioquinol suppresses EC angiogenesis by reducing VEGFR2 expression. Based on these results, we further compared the expression levels of VEGFR2 in HUVECs, HDMECs, hPC-PLs, NHDFs, MCF-7, MDA-MB-231, and 4T1-Luc2 cells. By means of Western blotting, we observed that VEGFR2 is highly expressed in both HUVECs and HDMECs, while its expression is rare in the other cell types (Fig. 2i, j). In addition, Pearson correlation analysis was performed to explore the association between the viability of different cell types exposed to clioquinol (Fig. 1a) and their VEGFR2 protein levels (Fig. 2i, j). This analysis demonstrated a strong negative correlation between cell viability and VEGFR2 expression following exposure to 10 or 25 µM clioquinol (Fig. 2k). Taken together, these findings suggest that clioquinol selectively inhibits the angiogenic activity of ECs predominantly by down-regulating VEGFR2.

### Clioquinol promotes VEGFR2 degradation

**Fig. 3:**
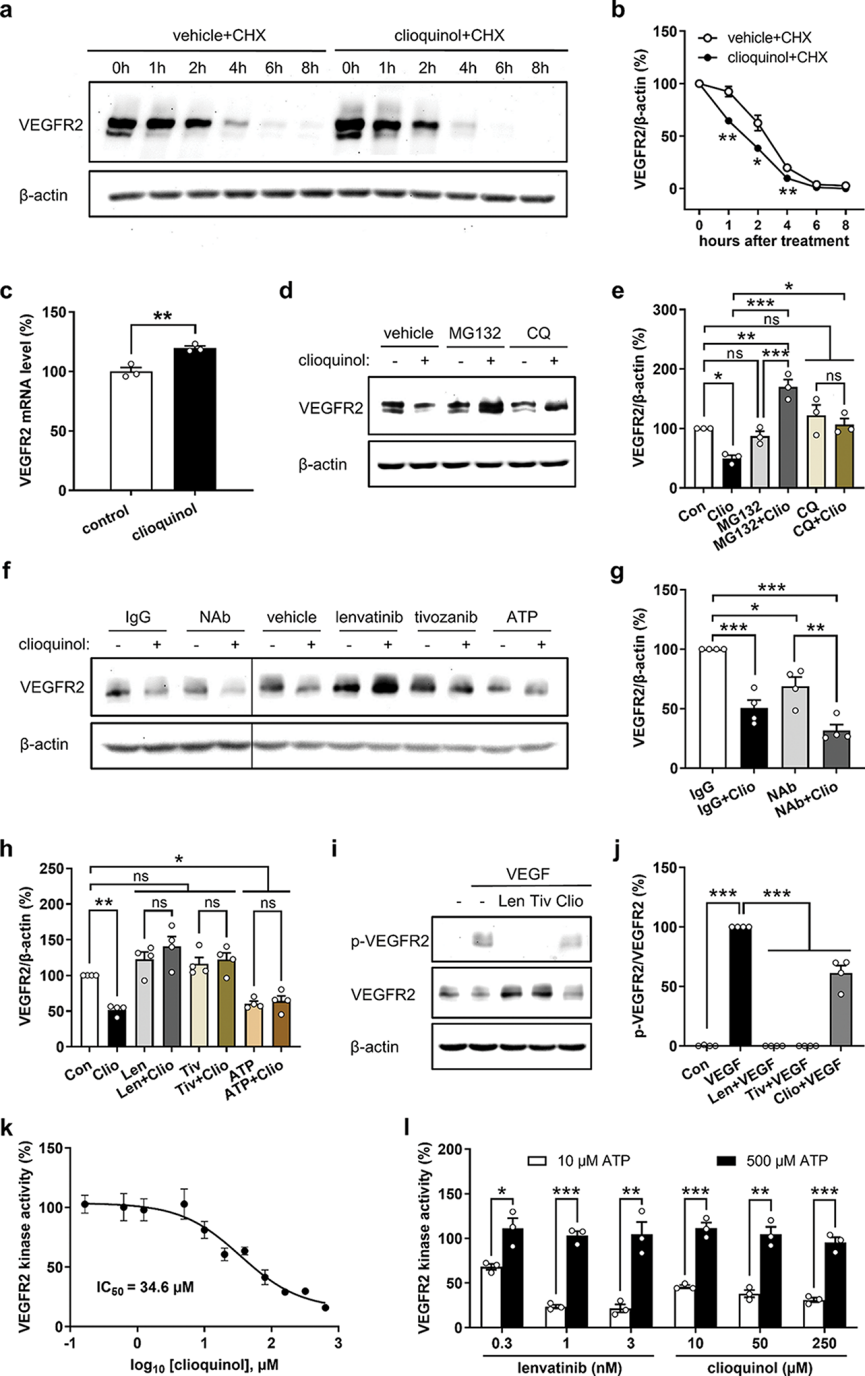
Clioquinol binds to the ATP-binding pocket of VEGFR2 and causes its degradation. **a** Western blots showing VEGFR2 and β-actin expression in HUVECs treated with 0.1% DMSO (vehicle) or 10 µM clioquinol in the presence of 100 µM cycloheximide (CHX) for 0, 1, 2, 4, 6, and 8 h. **b** Expression level (% of 0 h) of VEGFR2 normalized to β-actin in HUVECs treated as described in (**a**) (*n* = 3 independent experiments). **c** mRNA level of VEGFR2 (% of control) in HUVECs treated with 0.1% DMSO (control) or 10 µM clioquinol for 4 h, as assessed by real-time PCR (*n* = 3). **d** Western blots showing VEGFR2 and β-actin expression in HUVECs that were pre-treated without or with 20 µM MG132 or 200 µM chloroquine (CQ) for 2 h and then exposed to 0.1% DMSO or 10 µM clioquinol for another 4 h. **e** Expression level (% of control) of VEGFR2 normalized to β-actin in HUVECs treated as described in (d) (*n* = 3 independent experiments). **f** Western blots showing VEGFR2 and β-actin expression in HUVECs that were pre-treated with 4 µg/mL IgG, 4 µg/mL anti-VEGFR2 NAb, 0.1% DMSO (vehicle), 100 nM lenvatinib, 250 nM tivozanib, or 1 mM ATP for 2 h and then exposed to 0.1% DMSO or 10 µM clioquinol for another 4 h. **g**, **h** Expression level (% of IgG or control) of VEGFR2 normalized to β-actin in HUVECs treated as described in (f) (*n* = 4 independent experiments). **i** Western blots showing p-VEGFR2, VEGFR2, and β-actin expression in HUVECs that were treated with 0.1% DMSO, 100 nM lenvatinib, 250 nM tivozanib or 10 µM clioquinol for 1 h and then stimulated with 25 ng/mL VEGF for 7 min. **j** Expression level (% of control) of p-VEGFR2 normalized to VEGFR2 in HUVECs treated as described in (i) (*n* = 4 independent experiments). **k** VEGFR2 kinase activity (% of control) in the presence of serial dilutions of clioquinol at an ATP concentration of 10 µM, as assessed by VEGFR2 kinase assay (*n* = 2). **l** VEGFR2 kinase activity (% of control) in the presence of lenvatinib (0.3, 1, and 3 nM) or clioquinol (10, 50, and 250 µM) at ATP concentrations of 10 or 500 µM, as assessed by VEGFR2 kinase assay (*n* = 3). Means ± SEM. *P < 0.05, **P < 0.01, ***P < 0.001; ns, not significant. (e, g, h, j: one-way ANOVA with Tukey’s multiple comparisons test; b, c, l: unpaired Student’s t-test)

To investigate how clioquinol down-regulates VEGFR2 in ECs, we first examined VEGFR2 degradation in HUVECs treated with the vehicle DMSO or 10 µM clioquinol in the presence of the protein synthesis inhibitor cycloheximide (CHX). Western blot analyses revealed a gradual degradation of VEGFR2 over time in vehicle + CHX-treated HUVECs, reflecting the natural turnover of the VEGFR2 protein (Fig. 3a, b). Clioquinol treatment significantly accelerated this degradation process, with marked reductions observed at 1, 2, and 4 h post-treatment (Fig. 3a, b). These findings highlight the potent role of clioquinol as a VEGFR2 degrader.

To further exclude the possibility that clioquinol inhibits VEGFR2 transcription, we measured the mRNA levels of VEGFR2 in HUVECs treated with the vehicle DMSO or 10 µM clioquinol for 4 h. Of interest, real-time PCR analyses revealed that clioquinol even slightly increases VEGFR2 mRNA expression, suggesting the involvement of a negative feedback loop (Fig. 3c).

In eukaryotic cells, the protein degradation systems mainly consist of the proteasome and lysosome pathways [34]. To identify the system responsible for clioquinol-induced VEGFR2 degradation, HUVECs were pre-treated for 2 h with either the proteasome inhibitor MG132 or the lysosome inhibitor chloroquine (CQ) before exposure to clioquinol. Western blot analyses revealed that both MG132 and CQ completely reversed the clioquinol-induced degradation of VEGFR2 (Fig. 3d, e). This suggests that clioquinol promotes VEGFR2 degradation through both proteasome and lysosome systems.

### Clioquinol binds to the ATP-binding pocket of VEGFR2

In the chemical structure of clioquinol, there is a quinoline moiety (Supplementary Fig. 2a). Interestingly, lenvatinib and tivozanib, two well-known FDA-approved VEGFR2 inhibitors, also exhibit this ATP mimetic moiety (Supplementary Fig. 2a), enabling their binding to the ATP binding site of VEGFR2 [35, 36]. This led us to hypothesize that clioquinol may directly bind to the ATP-binding pocket of VEGFR2, thereby promoting its degradation.

We first performed molecular docking simulations to explore the potential binding modes of clioquinol within VEGFR2. For this purpose, lenvatinib, tivozanib, and ATP were used as reference molecules. Our results suggest that, similar to lenvatinib, tivozanib, and ATP, clioquinol preferentially binds to the ATP-binding pocket of VEGFR2 by forming a hydrogen bond with the Glu885 residue in the C-helix (Supplementary Fig. 2b).

To experimentally test this hypothesis, we pre-treated HUVECs for 2 h with either IgG or a neutralizing antibody (NAb) against VEGFR2 followed by clioquinol treatment. Of note, this NAb was generated using immunogen Ala20-Glu764, encompassing the extracellular domain and part of the transmembrane domain of VEGFR2. Western blot analyses showed that NAb slightly reduces VEGFR2 expression, but has no impact on clioquinol-induced VEGFR2 degradation (Fig. 3f, g). These results exclude the possibility that clioquinol directly interacts with the NAb’s binding region on the extracellular domain of VEGFR2. Furthermore, we utilized lenvatinib, tivozanib, and ATP to block the ATP-binding site of VEGFR2 on ECs prior to clioquinol treatment. For this purpose, non-cytotoxic doses of lenvatinib at 100 nM and tivozanib at 250 nM were chosen based on LDH assays (Supplementary Fig. 3a, b). ATP at 1 mM was employed, as demonstrated in a previous study [27], which significantly elevated intracellular ATP levels in HUVECs by 112% (Supplementary Fig. 3c). Western blot analyses showed that in the presence of lenvatinib, tivozanib, or ATP, clioquinol failed to induce VEGFR2 degradation (Fig. 3f, h). Of note, lenvatinib and tivozanib alone displayed no significant effects on VEGFR2 expression, while ATP caused a significant reduction (Fig. 3f, h). These findings indicate that clioquinol may bind to the ATP-binding site of VEGFR2, leading to its degradation.

In addition, we compared the effects of lenvatinib, tivozanib, and clioquinol on VEGFR2 phosphorylation after a short-term treatment of 1 h. As expected, lenvatinib and tivozanib completely inhibited the phosphorylation of VEGFR2 triggered by VEGF (Fig. 3i, j). Of interest, clioquinol significantly reduced VEGF-stimulated VEGFR2 phosphorylation only by 39% (Fig. 3i, j). Consistent with this observation, cell-free VEGFR2 kinase assays demonstrated that clioquinol exhibits a relatively weak inhibitory effect on VEGFR2 activity, with an IC_50_ of 34.6 µM at an ATP concentration of 10 µM (Fig. 3k). Importantly, the inhibitory effects of clioquinol at 10, 50, and 250 µM were completely reversed by the presence of 500 µM ATP, as shown by additional ATP competition experiments with lenvatinib serving as a positive control (Fig. 3l). These results provide strong evidence that clioquinol interacts directly with the ATP-binding site of VEGFR2.

### Clioquinol inhibits ERK phosphorylation and promotes AKT phosphorylation

**Fig. 4:**
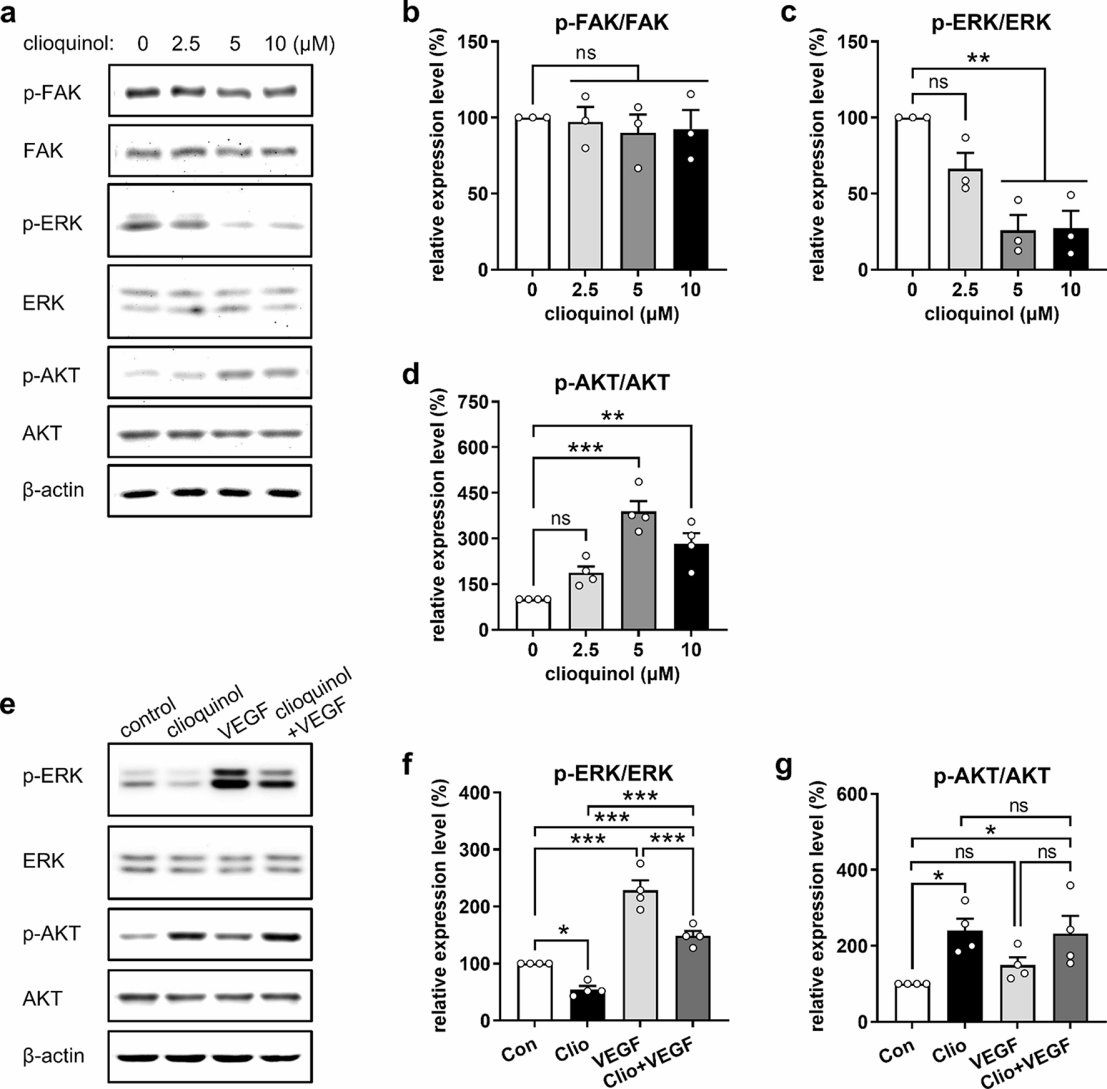
Clioquinol inhibits ERK phosphorylation while promoting AKT phosphorylation in ECs. **a** Western blots showing p-FAK, FAK, p-ERK, ERK, p-AKT, AKT, and β-actin expression in HUVECs after 4-hour treatment with 0, 2.5, 5, or 10 µM clioquinol. **b**-**d** Expression level (% of 0 µM) of p-FAK normalized to FAK (b), p-ERK normalized to ERK (c), and p-AKT normalized to AKT (d) in HUVECs treated as described in (a) (*n* = 3–4 independent experiments). **e** Western blots showing p-ERK, ERK, p-AKT, AKT, and β-actin expression in HUVECs that were treated with 0.1% DMSO (control) or 10 µM clioquinol for 4 h and then stimulated with 25 ng/mL VEGF for 7 min. **f**, **g** Expression level (% of control) of p-ERK normalized to ERK (f) and p-AKT normalized to AKT (g) in HUVECs treated as described in (**e**) (*n* = 4 independent experiments). Means ± SEM. *P < 0.05, **P < 0.01, ***P < 0.001; ns, not significant. (b-**d**, **f**, **g**: one-way ANOVA with Tukey’s multiple comparisons test)

To further elucidate the downstream signaling pathways responsible for the anti-angiogenic effects of clioquinol, we assessed the phosphorylation levels of FAK, ERK, and AKT in HUVECs exposed to different concentrations of clioquinol for 4 h. Western blot analyses demonstrated that 5 and 10 µM clioquinol significantly inhibits ERK phosphorylation while, unexpectedly, promotes AKT phosphorylation with no impact on FAK phosphorylation (Fig. 4a-d). Subsequent administration of VEGF to clioquinol-treated HUVECs rescued the phosphorylation of ERK suppressed by 10 µM clioquinol but had no effect on clioquinol-induced AKT phosphorylation (Fig. 4e-g). These findings indicate that the anti-angiogenic effects of clioquinol are primarily mediated by down-regulation of ERK phosphorylation, which is at least partially mediated by VEGFR2 degradation.

### Clioquinol and AKT inhibitor synergistically inhibit EC angiogenesis

**Fig. 5:**
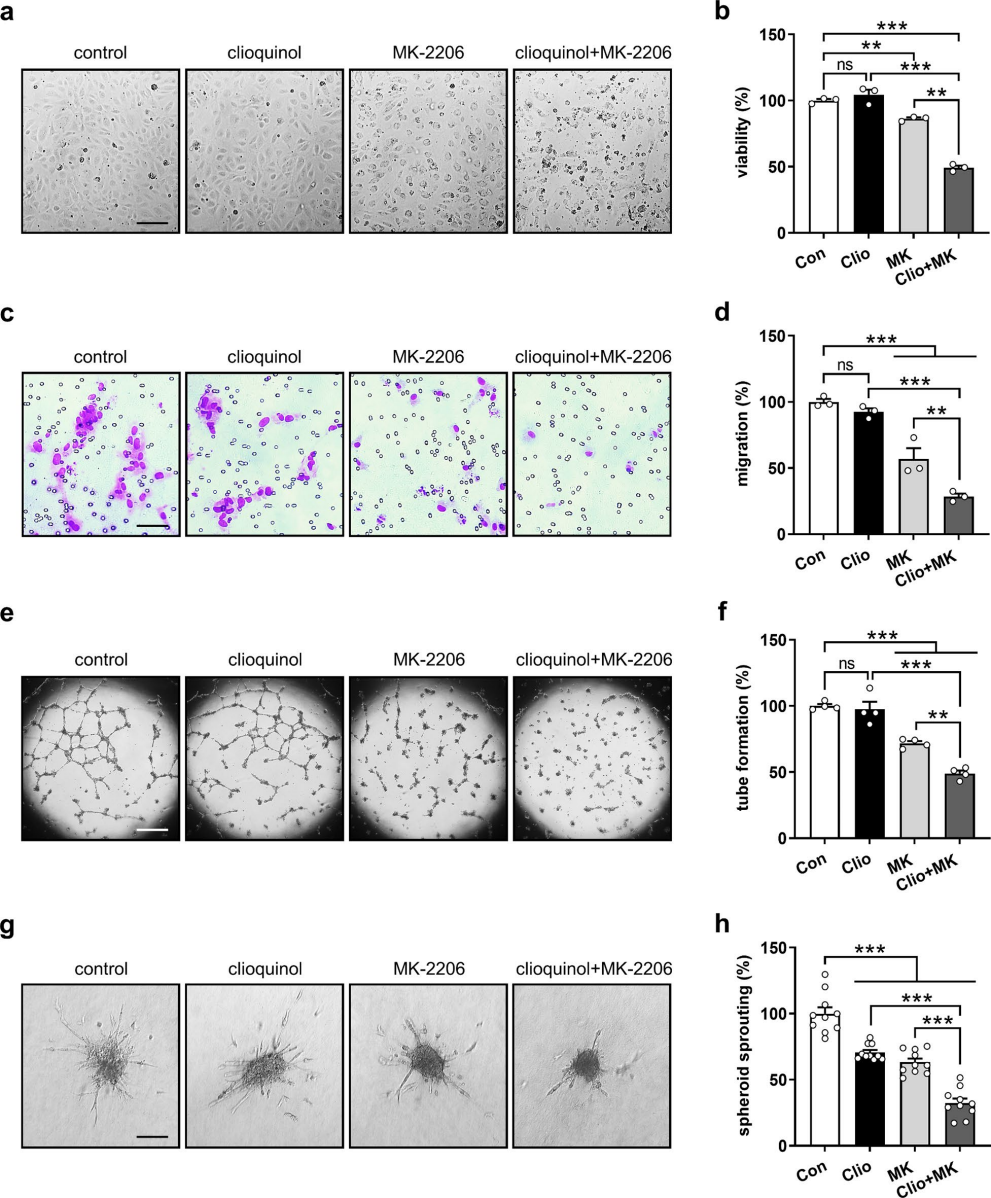
Clioquinol and MK-2206 synergistically inhibit EC angiogenesis. **a** Light microscopic images of HUVECs treated with 0.1% DMSO or 2.5 µM clioquinol in the absence or presence of 5 µM MK-2206 for 48 h. Scale bar: 145 μm. **b** Viability (% of control) of HUVECs treated as described in (a) (*n* = 3). **c** Light microscopic images of migrated HUVECs after 5-hour incubation. The cells were treated with 0.1% DMSO or 2.5 µM clioquinol in the absence or presence of 5 µM MK-2206 for 24 h prior to this assay. Scale bar: 65 μm. **d** Migration (% of control) of HUVECs treated as described in (c) (*n* = 3). **e** Phase-contrast microscopic images of tube-forming HUVECs after 18-hour treatment with 0.1% DMSO or 2.5 µM clioquinol in the absence or presence of 5 µM MK-2206. Scale bar: 700 μm. **f** Tube formation (% of control) of HUVECs treated as described in (e) (*n* = 4). **g** Phase-contrast microscopic images of HUVEC spheroids after 24-hour treatment with 0.1% DMSO or 2.5 µM clioquinol in the absence or presence of 5 µM MK-2206. Scale bar: 135 μm. **h** Sprouting (% of control) of HUVEC spheroids treated as described in (**g**) (*n* = 10). Means ± SEM. **P < 0.01, *** *P* < 0.001; ns, not significant. (b, d, f, h: one-way ANOVA with Tukey’s multiple comparisons test)

The AKT pathway is essential for angiogenesis, facilitating EC survival, proliferation, migration, and tube formation, whereas its hyperactivation has been implicated as a significant contributor to drug resistance [37, 38]. We thus hypothesized that combining clioquinol with the AKT inhibitor MK-2206 could enhance its anti-angiogenic efficacy. To test this hypothesis, we chose a dose of 2.5 µM clioquinol, which alone had no effect on HUVEC viability, migration, and tube formation (Fig. 5a-f). In parallel, 5 µM MK-2206 alone moderately suppressed these angiogenic processes (Fig. 5a-f). However, the combination of 2.5 µM clioquinol and 5 µM MK-2206 exhibited a much stronger synergistic inhibitory effect (Fig. 5a-f). This was further supported by the results of spheroid sprouting assays (Fig. 5g, h).

In an additional set of experiments, we investigated the effects of clioquinol, MK-2206, and their combination on the viability of 4T1 cells. WST-1 assays revealed that 2.5 µM clioquinol alone has no impact on 4T1 cell viability, while 5 µM MK-2206 alone slightly reduces cell viability by 4%. The combination of both compounds resulted in a similar effect compared to MK-2206 alone (Supplementary Fig. 4). These findings suggest that 2.5 µM clioquinol and 5 µM MK-2206 have no synergistic inhibitory effect on 4T1 cell viability, demonstrating much lower sensitivity of 4T1 cells to these compounds when compared to HUVECs.

### Clioquinol and MK-2206 inhibit TNBC angiogenesis and growth

**Fig. 6:**
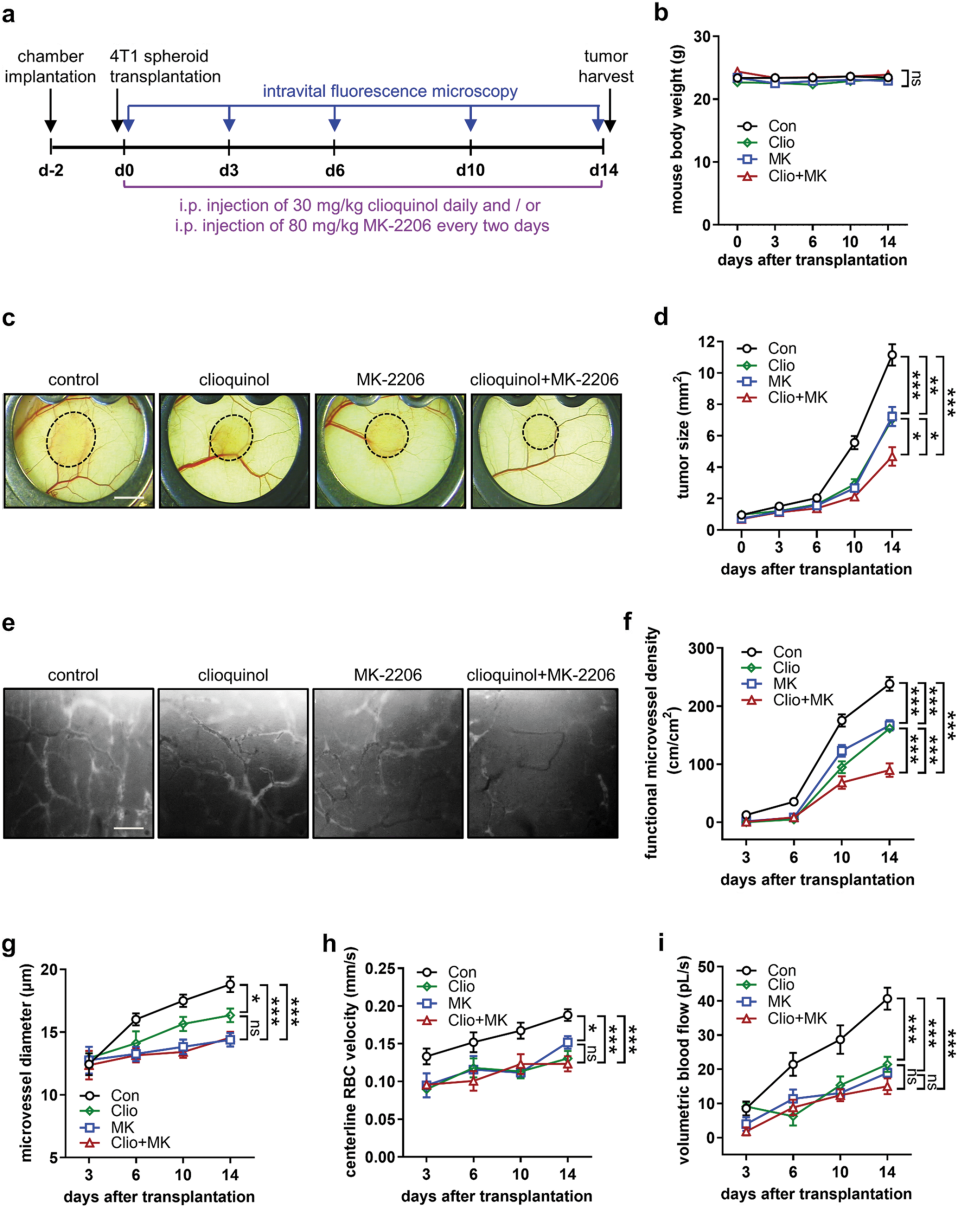
Clioquinol and MK-2206 inhibit TNBC development, as assessed by intravital fluorescence microscopy. **a** Timeline of the experiments in the dorsal skinfold chamber model. **b** Body weight (g) of mice in control, clioquinol, MK-2206, and combination group on days 0, 3, 6, 10, and 14 after tumor transplantation (*n* = 10). **c** Stereomicroscopic images of 4T1 tumors (bordered by broken line) in mice from control, clioquinol, MK-2206, and combination group on day 14 after tumor transplantation. Scale bar: 2 mm.**d** Tumor size (mm^2^) in control, clioquinol, MK-2206, and combination group on days 0, 3, 6, 10, and 14 after tumor transplantation, as assessed by intravital fluorescence microscopy (*n* = 10). **e** Intravital fluorescence microscopic images of tumor microvessels in control, clioquinol, MK-2206, and combination group on day 14 after tumor transplantation. Scale bar: 120 μm. **f** Functional microvessel density (cm/cm^2^) of 4T1 tumors in control, clioquinol, MK-2206, and combination group on days 0, 3, 6, 10, and 14 after tumor transplantation, as assessed by intravital fluorescence microscopy (*n* = 10). **g**-**i** Diameter (µm; g), centerline RBC velocity (mm/s; h), and volumetric blood flow (pL/s; i) of tumor microvessels in control, clioquinol, MK-2206, and combination group, as assessed by intravital fluorescence microscopy (*n* = 10). Means ± SEM. *P < 0.05, **P < 0.01, ***P < 0.001; ns, not significant. (b, d, f, g-i: one-way ANOVA with Tukey’s multiple comparisons test)

We subsequently evaluated the combined effects of clioquinol and MK-2206 on TNBC development in a dorsal skinfold chamber model by means of intravital fluorescence microscopy, as illustrated in the timeline shown in Fig. 6a. Daily administration of 30 mg/kg body weight clioquinol, every two-day administration of 80 mg/kg body weight MK-2206, or their combination over a period of 14 days had no significant impact on the body weight of the treated mice (Fig. 6b). The treated animals also showed typical activity patterns comparable to those observed in controls. However, treatment with clioquinol or MK-2206 alone significantly decreased the size of 4T1 tumors (Fig. 6c, d) as well as the density of functional blood-perfused microvessels on days 10 and 14 after tumor transplantation (Fig. 6e, f). Importantly, the combination of clioquinol with MK-2206 outperformed the efficacy of the single treatments on day 14 after tumor transplantation (Fig. 6c-f). The CDI value of 0. 79 in Fig. 6f indicates a synergistic effect of these two compounds in reducing tumor vessel density (Supplementary Table 1). The potent combined effect of clioquinol and MK-2206 was further confirmed by means of bioluminescence imaging (Supplementary Fig. 5). Additional microhemodynamic analyses revealed a significantly reduced diameter, centerline RBC velocity, and volumetric blood flow of tumor microvessels in the clioquinol, MK-2206, and combination groups, compared to control group, on days 10 and 14 after tumor transplantation (Fig. 6g-i).

**Fig. 7:**
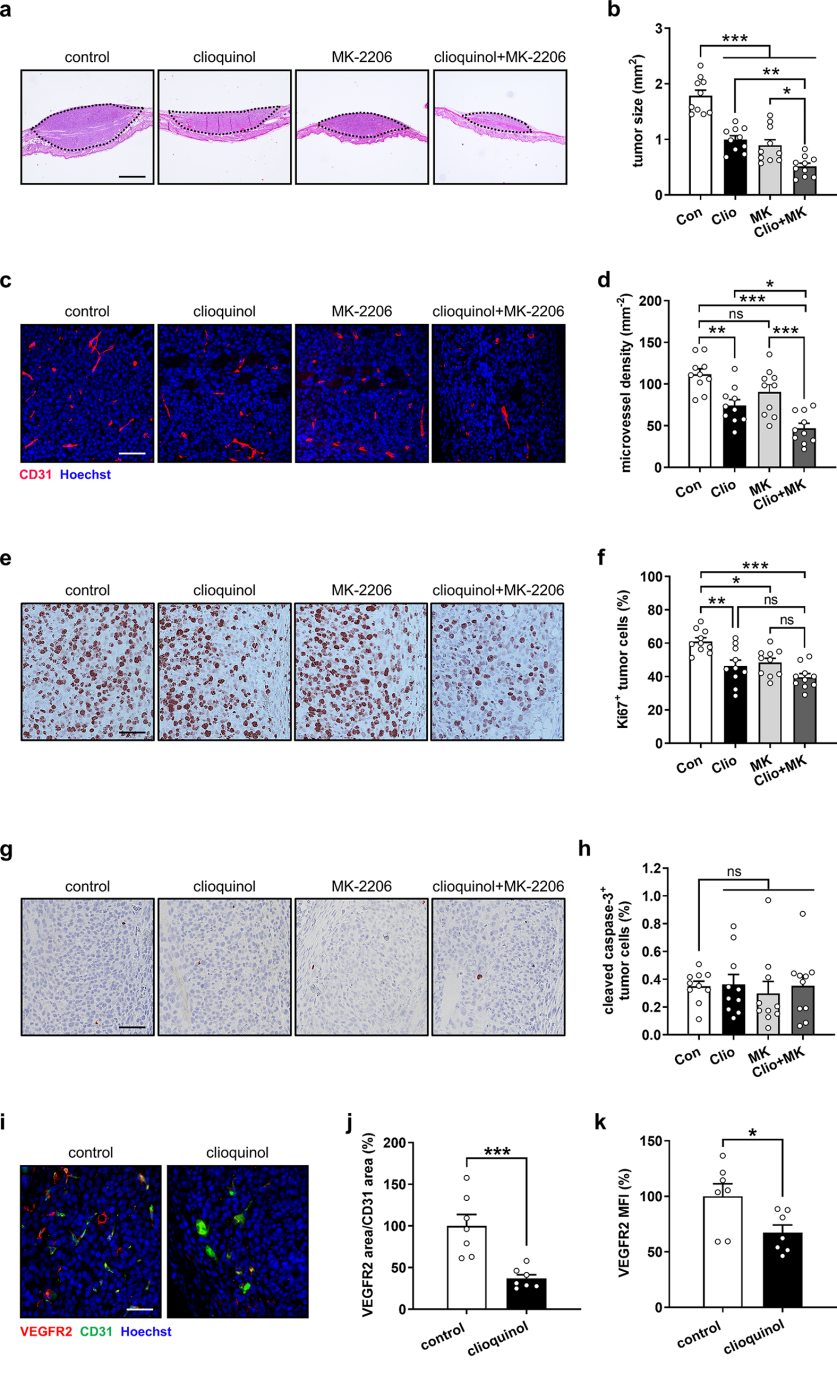
Clioquinol and MK-2206 inhibit TNBC development, as assessed by histology and immunohistochemistry. **a** Light microscopic images of H&E-stained 4T1 tumors (bordered by dotted line) in control, clioquinol, MK-2206, and combination group. Scale bar: 160 μm. **b** Tumor size (mm^2^) in control, clioquinol, MK-2206, and combination group, as assessed by histology (*n* = 10). **c** Fluorescence microscopic images of tumor microvessels in control, clioquinol, MK-2206, and combination group. Tumor sections were stained with an anti-CD31 antibody (red) and Hoechst 33342 (blue) for the visualization of ECs and cell nuclei, respectively. Scale bar: 70 μm. **d** Microvessel density (mm^− 2^) of 4T1 tumors in control, clioquinol, MK-2206, and combination group, as assessed by immunohistochemistry (*n* = 10). **e**, **g** Light microscopic images of Ki67- (e) and cleaved caspase-3-positive (g) tumor cells in control, clioquinol, MK-2206, and combination group. Scale bars: 55 μm. **f**, **h** Ki67- (f) and cleaved caspase-3-positive tumor cells (h) (% of total cell number) in control, clioquinol, MK-2206, and combination group, as assessed by immunohistochemistry (*n* = 10). **i** Fluorescence microscopic images of tumor microvessels in control and clioquinol group. Tumor sections were stained with an anti-VEGFR2 antibody (red), an anti-CD31 antibody (green), and Hoechst 33342 (blue). Scale bar: 60 μm. **j** Area of VEGFR2 signal normalized to CD31 area (% of control) in tumors of control and clioquinol group. (k) VEGFR2 MFI (% of control) in tumors of control and clioquinol group. Means ± SEM. *P < 0.05, **P < 0.01, ***P < 0.001; ns, not significant. (b, d, f, h: one-way ANOVA with Tukey’s multiple comparisons test; j, k: unpaired Student’s t-test)

Finally, histological and immunohistochemical analyses showed that tumors in clioquinol- and MK-2206-treated mice exhibited smaller sizes when compared to those in control animals (Fig. 7a, b). The combination of these two compounds demonstrated an even stronger inhibition of tumor growth (Fig. 7a, b). Moreover, clioquinol significantly reduced the density of tumor microvessels, while MK-2206 showed no effect. However, their combination synergistically suppressed tumor angiogenesis with a CDI value of 0.78 (Fig. 7c, d; Supplementary Table 1). In addition, clioquinol, MK-2206, and their combination markedly decreased the percentage of Ki67-positive proliferating tumor cells (Fig. 7e, f) without affecting the fraction of cleaved caspase-3-positive apoptotic tumor cells (Fig. 7g, h). Notably, no morphological evidence of tumor cell necrosis was detected in any group. In addition, we assessed the expression of VEGFR2 in tumors of clioquinol-treated and control mice by immunohistochemical double staining of VEGFR2 and CD31. Our results revealed that clioquinol effectively reduced the area of VEGFR2 signal (normalized to the area of CD31 signal) as well as its intensity (Fig. 7i-k).

## Discussion

Despite its historical association with SMON, clioquinol showed no toxicity in clinical trials for Alzheimer’s disease [39, 40]. This has encouraged researchers to explore repurposing of this agent for cancer therapy. Accordingly, previous studies have extensively analyzed the anti-proliferative and cytotoxic effects of clioquinol on multiple types of tumor cells [15–17, 41–44]. Mechanistically, clioquinol acts as a chelator or ionophore of divalent metals, such as copper and zinc, causing proteasome inhibition or lysosome disruption in cancer cells. For instance, as a zinc ionophore, 30–45 µM clioquinol induced apoptosis of human B-cell lymphoma Raji cells after treatment for 24 h [15]. A 48-hour treatment with 10–30 µM clioquinol reduced the viability of leukemia and myeloma cells, possibly by inhibiting the proteasome [17]. Similarly, 24-hour treatment with 50 µM clioquinol triggered proteasome inhibition and apoptosis in CuCl_2_-pretreated human prostate cancer LNCaP and C4-2B cells [41]. In human prostate cancer DU 145 cells, a combination of 10 µM clioquinol and 50 µM ZnCl_2_ increased lysosome permeability and induced cytotoxicity [42, 43]. Additionally, a recent study reported that treatment with 20 µM clioquinol for 24 h induces pyroptosis of leukemia and myeloma cells via up-regulating IFIT1 and IFIT3 [45]. Nevertheless, the potential impact of clioquinol on ECs remains unexplored. This gap of knowledge is closed now by the present study, which demonstrates for the first time that clioquinol effectively inhibits the angiogenic activity of ECs through promoting VEGFR2 degradation. Additionally, it boosts the effectiveness of the AKT inhibitor MK-2206 in suppressing the vascularization and growth of TNBC.

The TME is a highly heterogeneous and complex system containing cancer cells, ECs, pericytes, fibroblasts, immune cells, and other cell types [46]. Each cell type of the TME plays a critical role in supporting tumor development and progression [46]. For instance, pericytes have been reported to trigger tumor vessel dysfunction, therefore facilitating tumor metastasis and immune evasion [47–50]. In addition, fibroblasts, which are a predominant component of the tumor stroma, promote the vascularization, growth, and metastasis of tumors [51, 52]. Interestingly, we herein observed that both types of examined ECs, i.e. HUVECs and HDMECs, exhibit a markedly higher sensitivity to clioquinol when compared to breast cancer cells, pericytes, and fibroblasts. In vitro and in vivo angiogenesis assays further revealed a potent inhibitory effect of clioquinol on the angiogenic activity of ECs. These findings suggest that clioquinol preferentially targets ECs within a tumor and that the inhibition of angiogenesis primarily contributes to the anti-cancer properties of clioquinol.

Based on these assumptions, we additionally investigated the molecular mechanisms underlying the anti-angiogenic effects of clioquinol. Western blot analyses revealed that exposure to clioquinol for 4 h significantly and specifically promotes the degradation of VEGFR2 in ECs. However, unlike other VEGFR2 degraders synthesized via proteolysis-targeting chimera technology [53, 54], clioquinol acts via both the proteasome and lysosome degradation systems. VEGFR2 is the most important receptor on ECs, orchestrating VEGF-induced angiogenesis, as its activation mediates EC survival, proliferation, migration and enhances vascular permeability [55]. Given this central role, ECs are likely highly sensitive to changes in VEGFR2 levels. Thus, even slight increases in clioquinol concentration, which further promote VEGFR2 degradation, could rapidly impair cell viability, resulting in the unusually sharp dose-response curve observed for clioquinol in inhibiting EC viability. While predominantly expressed on ECs, VEGFR2 has also been reported to be expressed in a wide range of tumor cells, including breast cancer cells [56, 57]. However, in our experimental setting, VEGFR2 was exclusively highly expressed in ECs with a negligible expression in breast cancer cells, pericytes, and fibroblasts. The significant correlation between the viability of these cells in response to clioquinol and their VEGFR2 expression indicates that VEGFR2 targeting contributes to the high selectivity of clioquinol against ECs.

Further mechanistic analyses showed that clioquinol directly binds to the ATP-binding site of VEGFR2, as evidenced by the observation that pre-treatment with lenvatinib, tivozanib, or ATP completely blocked clioquinol-induced VEGFR2 degradation. In addition, brief exposure to clioquinol for 1 h significantly reduced VEGF-induced VEGFR2 phosphorylation. Therefore, despite directly interacting with the ATP-binding site of VEGFR2 like lenvatinib and tivozanib, clioquinol exhibits a unique impact on VEGFR2 regulation. In fact, as a short-term effect clioquinol moderately inhibited VEGFR2 phosphorylation, whereas it ultimately and strongly promoted VEGFR2 degradation. Therefore, we assume that although inhibition of VEGFR2 phosphorylation may contribute to clioquinol’s anti-angiogenic effects, the more profound impact on EC angiogenesis rather results from clioquinol-induced VEGFR2 degradation. This mode of action has the potential to prevent tumor resistance and enhance treatment outcomes [58, 59], making clioquinol a promising alternative to traditional small-molecule VEGFR2 kinase inhibitors, such as lenvatinib and tivozanib. For this, the clioquinol-VEGFR2 binding interaction should be further analyzed with techniques such as X-ray crystallography. This may clarify how clioquinol changes the conformation of VEGFR2, rendering this receptor susceptible to degradation.

In another set of experiments, we studied the effects of clioquinol on the two major VEGFR2 downstream kinases ERK and AKT that trigger the angiogenic activity of ECs. Of interest, treatment of ECs with clioquinol resulted in a decrease of ERK phosphorylation but an increase of AKT phosphorylation. We further found that the down-regulation of the ERK pathway may be related to the clioquinol-induced degradation of VEGFR2, which was not the case for the up-regulation of the AKT pathway. The increase in AKT phosphorylation may act as a compensatory or feedback response, even after a single treatment with clioquinol, enabling ECs to adapt to the stress caused by clioquinol-induced ERK dephosphorylation, potentially reducing its effectiveness. Therefore, it is assumed that the increase of AKT phosphorylation represents a resistance mechanism in ECs against clioquinol. This view is supported by our observation that combining clioquinol with the AKT inhibitor MK-2206 was much more effective in inhibiting EC angiogenesis when compared to a single treatment with clioquinol. In fact, the AKT pathway has been implicated in the development of drug resistance in a variety of cancer types. Accordingly, the application of AKT inhibitors in conjunction with other therapeutic modalities, including chemotherapy, hormone therapy, radiotherapy, and immunotherapy, holds great promise in overcoming tumor resistance [37, 60, 61].

Finally, we analyzed the effects of clioquinol, either alone or in combination with MK-2206, on tumor angiogenesis and growth in a dorsal skinfold chamber model of TNBC. TNBC is defined by the lack of estrogen receptor, progesterone receptor, and HER2 [62]. We chose this type of cancer due to its classification as the most aggressive subtype of breast cancer with a poor prognosis and limited treatment options [62]. Furthermore, there have been no reports on the effects of clioquinol on TNBC development. For our experiments, we used murine 4T1 mammary cancer cells. This cell line shares crucial molecular features with human TNBC and, thus, is widely utilized in syngeneic murine TNBC models [63]. Our results demonstrated that daily intraperitoneal injections of 30 mg/kg body weight clioquinol significantly inhibit TNBC angiogenesis and growth. In a previous study, Ogata et al. reported a concentration of 4.2 µg/mL (approximately 13.8 µM) [^14^C]-clioquinol in mouse blood 24 h after an intraperitoneal injection of a 100 mg/kg dose [64]. Based on this finding, we estimate that a dose of 30 mg/kg clioquinol would yield blood concentrations around 4.2 µM. This estimated in vivo concentration aligns well with the effective concentrations used in our in vitro experiments (2.5–10 µM), supporting the plausibility of our mechanistic findings at achievable in vivo levels. The anti-angiogenic effect of clioquinol was even more pronounced when combining it with MK-2206. Therefore, it may be interesting to clarify in future studies whether other AKT inhibitors, such as the recently FDA-approved ATP-competitive inhibitor capivasertib, may also enhance the efficacy of clioquinol.

**Fig. 8:**
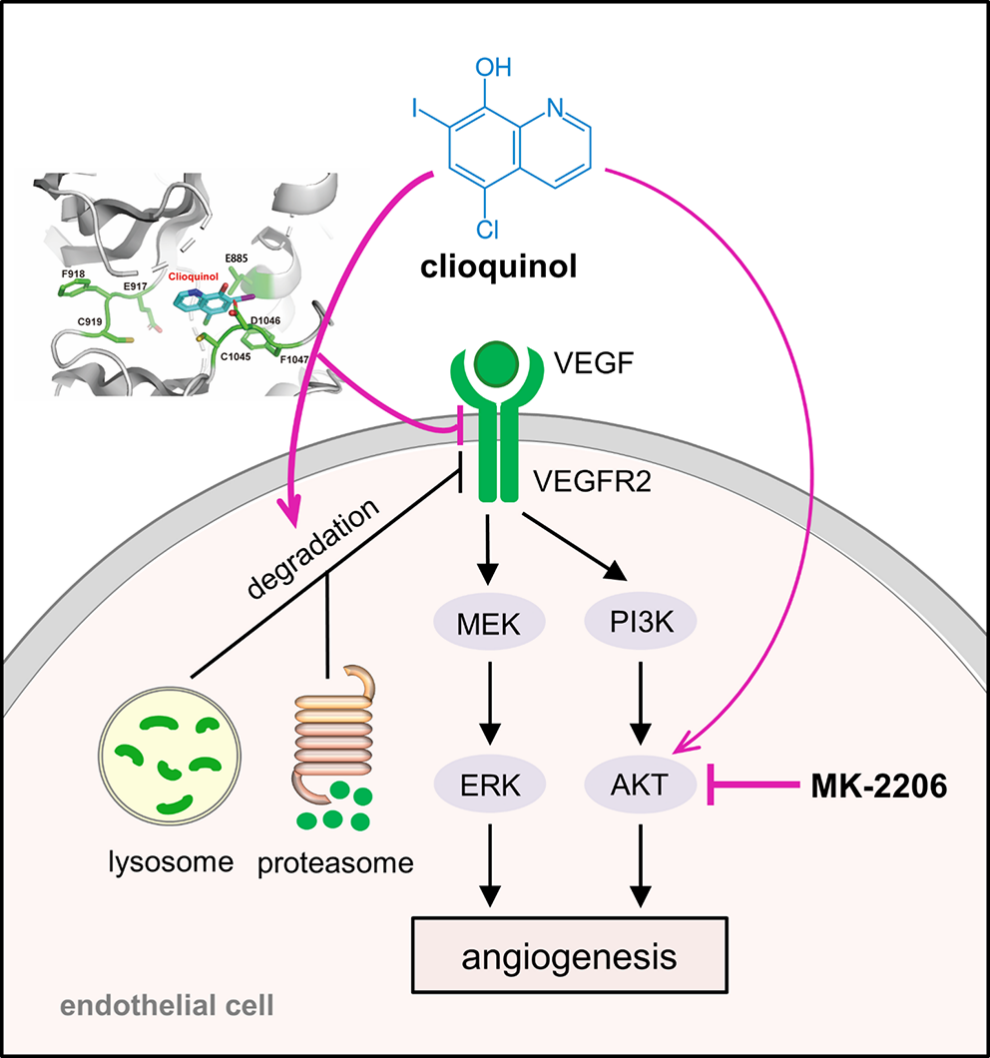
Scheme illustrating the molecular mechanisms underlying the potent inhibitory effects of clioquinol alone and its synergistic inhibitory effects with MK-2206 on angiogenesis. Clioquinol binds directly to the ATP-binding site of VEGFR2 on ECs, leading to a transient inhibition of VEGFR2 phosphorylation induced by VEGF and eventual promotion of VEGFR2 degradation via both the proteasome and lysosome systems. Consequently, the downstream ERK pathway is down-regulated. Furthermore, clioquinol increases AKT phosphorylation, while the inhibition of AKT by MK-2206 synergistically enhances the anti-angiogenic efficacy of clioquinol

In summary, our study demonstrates a potent anti-angiogenic effect of clioquinol, which is attributed to its action as an efficient VEGFR2 degrader (Fig. 8). Moreover, clioquinol exhibits synergistic effects with the AKT inhibitor MK-2206 in suppressing angiogenesis, resulting in the inhibition of TNBC growth. Therefore, clioquinol holds great promise for repurposing in future anti-angiogenic cancer therapy, whether administered alone or in combination with AKT inhibitors.

## Electronic Supplementary Material

Below is the link to the electronic supplementary material.

Supplementary Material 1

Supplementary Material 2

## Data Availability

No datasets were generated or analysed during the current study.
