# Supplementary material for: Clioquinol inhibits angiogenesis by promoting VEGFR2 degradation and synergizes with AKT inhibition to suppress triple-negative breast cancer vascularization
Source: Angiogenesis. 2025 Feb 3;28(2):13. doi: 10.1007/s10456-024-09965-1 (PMC11790708; doi:10.1007/s10456-024-09965-1)
Supplement: Supplementary file 1 — Supplementary Material 1 [file 10456_2024_9965_MOESM1_ESM.docx]

**Supplementary Material**

**Clioquinol inhibits angiogenesis by promoting VEGFR2 degradation and synergizes with AKT inhibition to suppress triple-negative breast cancer vascularization**

Yuan Gu^1^*, Tianci Tang^1^, Moqin Qiu^1,2^, Hongmei Wang^3^, Emmanuel Ampofo^1^, Michael D. Menger^1^, Matthias W. Laschke^1^

^1^Institute for Clinical and Experimental Surgery, Saarland University, 66421 Homburg, Germany

^2^Department of Respiratory Oncology, Guangxi Medical University Cancer Hospital, 530021 Nanning, China

^3^Shaanxi University of Chinese Medicine, 712046 Shaanxi, China

***Corresponding author:**

Yuan Gu, Ph.D.

Institute for Clinical and Experimental Surgery

Saarland University

66421 Homburg/Saar

Germany

phone: +49 6841 162 6368

fax: +49 6841 162 6553

e-mail: yuan.gu@uks.eu

**
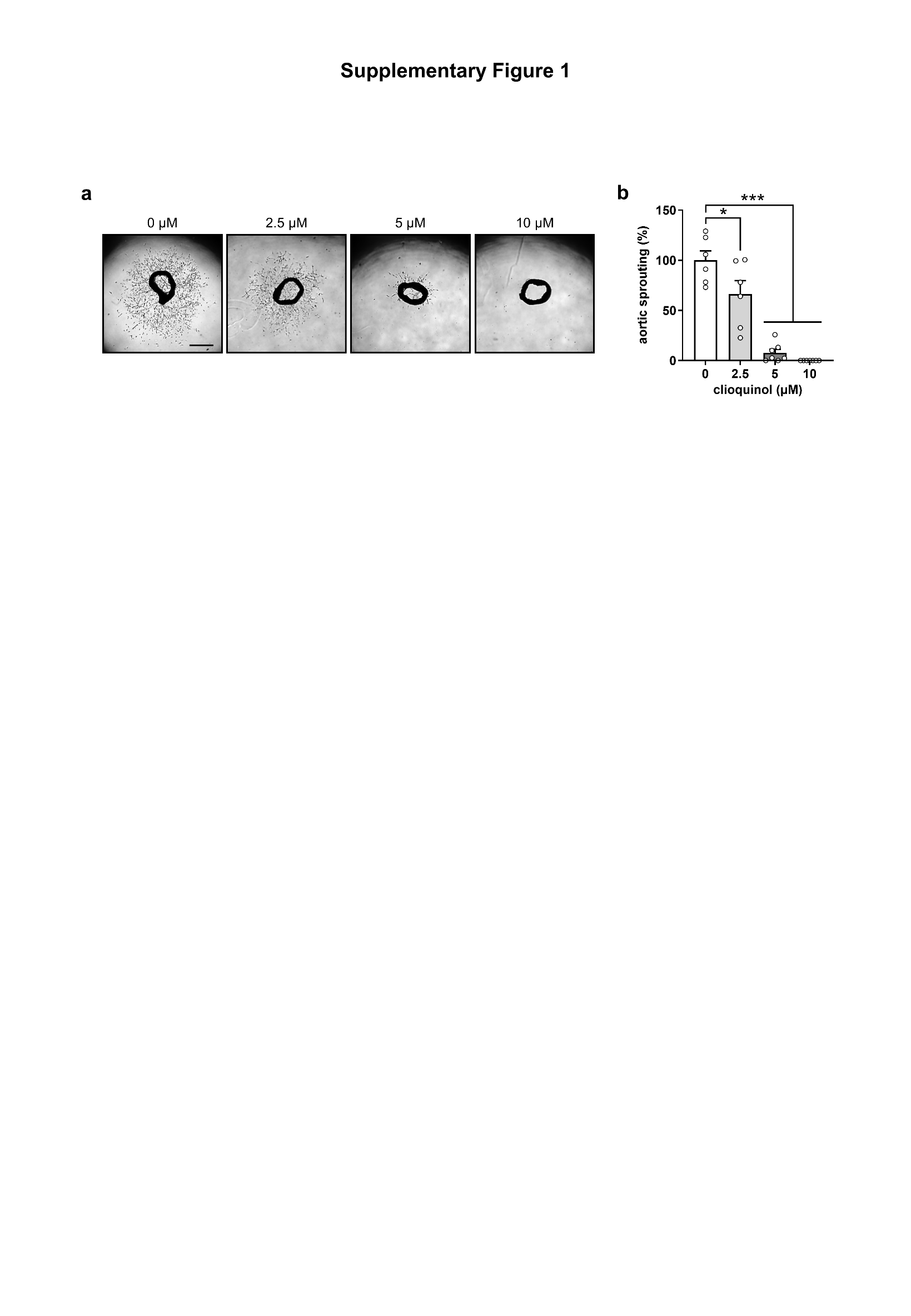
**

**Supplementary Fig. 1** Clioquinol inhibits aortic sprouting ex vivo. **a** Phase-contrast microscopic images of mouse aortic rings after 6-day treatment with 0, 2.5, 5 or 10 µM clioquinol. Scale bar: 630 µm. **b** Sprouting (% of 0 µM) of aortic rings treated as described in (a) (n = 6-7). Means ± SEM. *P < 0.05, ***P < 0.001. (b: one-way ANOVA with Tukey’s multiple comparisons test).


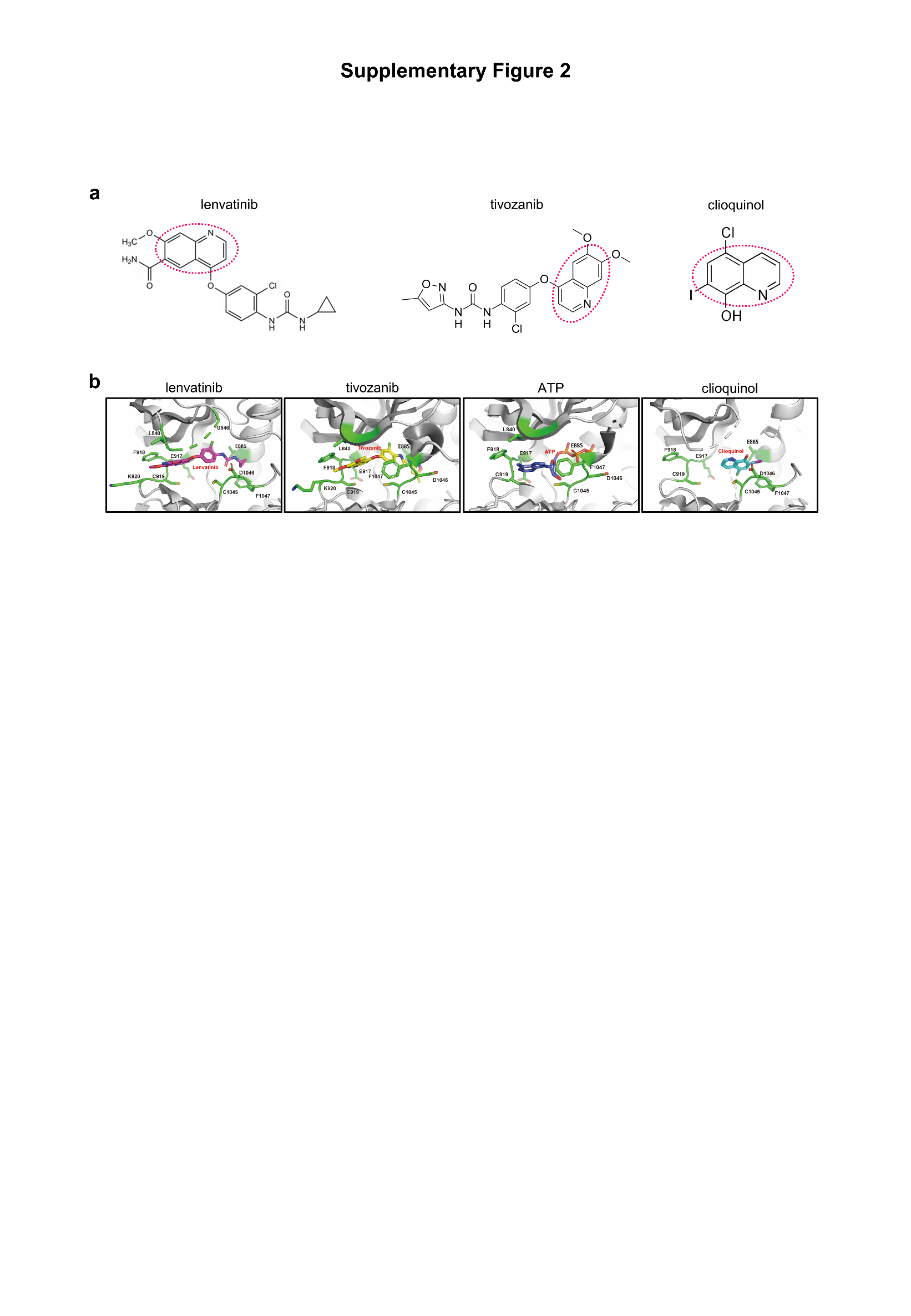


**Supplementary Fig. 2** Comparison of clioquinol with lenvatinib and tivozanib in structure and binding mode within VEGFR2. **a** Molecular structures of lenvatinib, tivozanib, and clioquinol. The quinoline moiety of each compound is highlighted with dotted circles. **b** 3D binding modes of VEGFR2 with lenvatinib (PDB ID: 3WZD), tivozanib (PDB ID: 4ASE), ATP, and clioquinol. Interaction sites within VEGFR2 are highlighted in green.


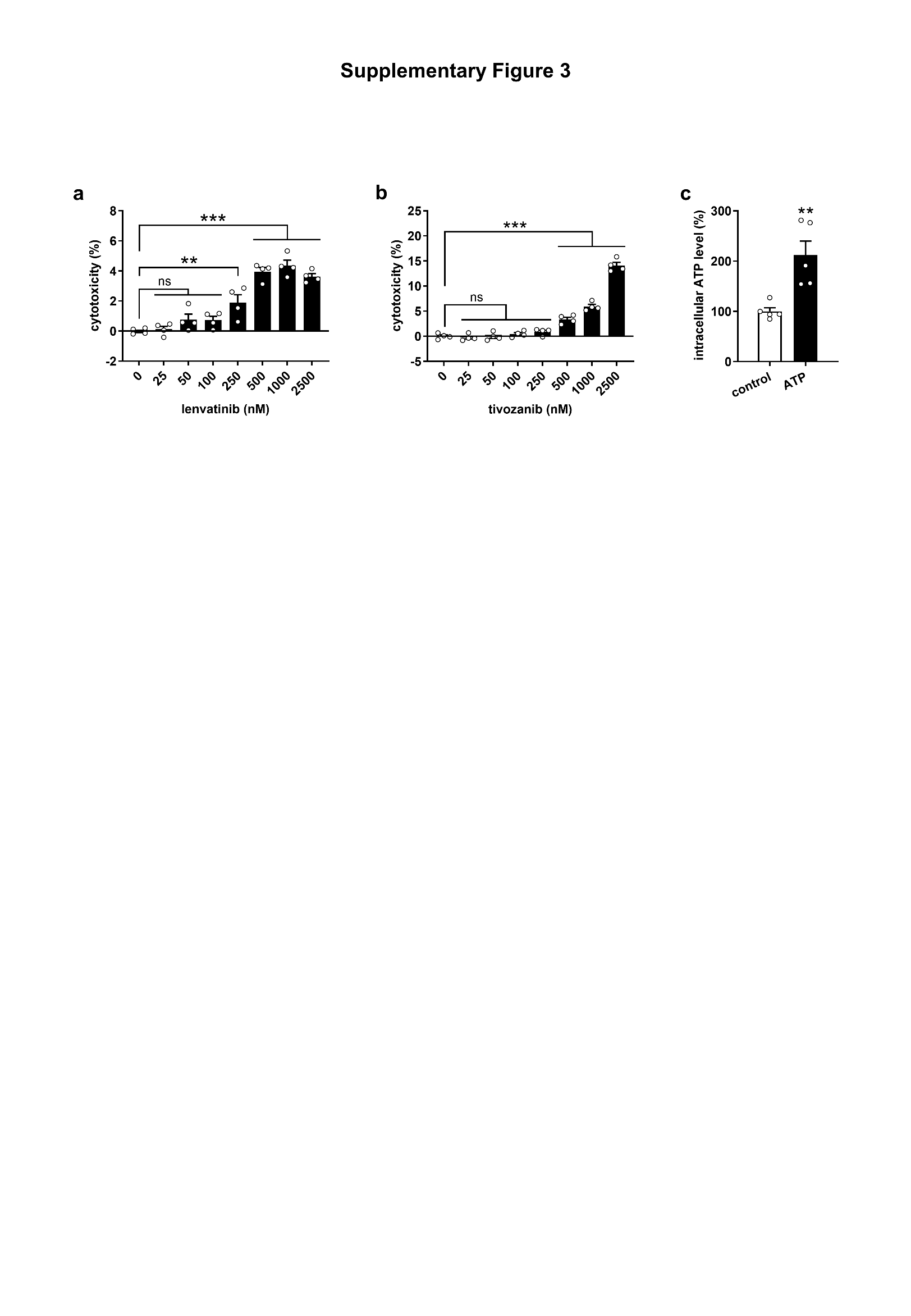


**Supplementary Fig. 3** Effects of lenvatinib, tivozanib, and ATP on HUVECs. **a**, **b** Cytotoxicity (% of total cell death) of lenvatinib (a) and tivozanib (b) against HUVECs after 24-hour treatment, as assessed by LDH assay (n = 4). **c** Intracellular ATP level (%) in HUVECs treated with or without 1 mM ATP for 2 hours, as assessed by luciferase ATP assays (n = 5). Means ± SEM. **P < 0.01, ***P < 0.001. (a, b: one-way ANOVA with Tukey’s multiple comparisons test; c: unpaired Student’s t-test).


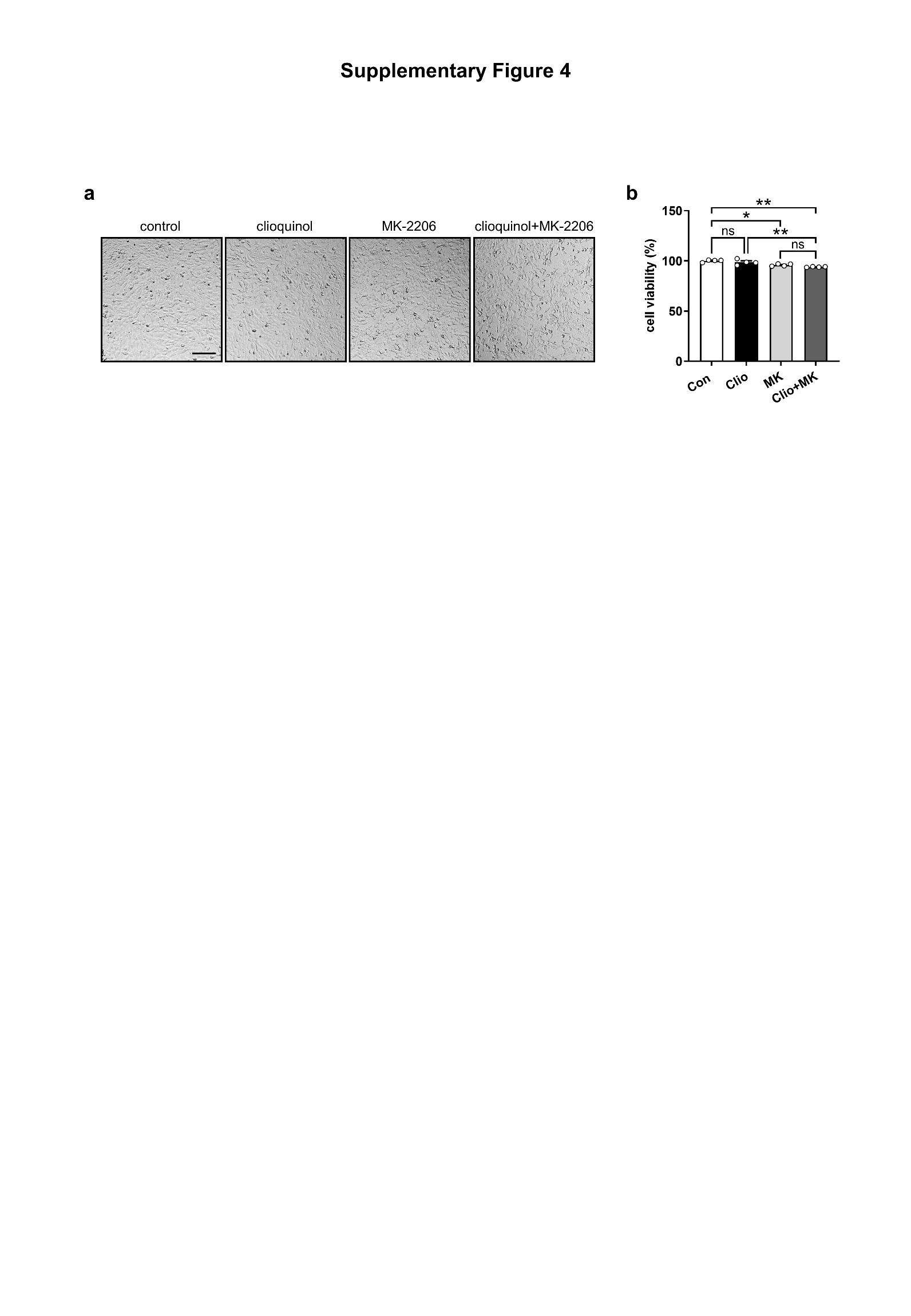


**Supplementary Fig. 4** Effects of clioquinol, MK-2206, and their combination on 4T1 cell viability. **a** Light microscopic images of 4T1-Luc2 cells treated with 0.1% DMSO or 2.5 µM clioquinol in the absence or presence of 5 µM MK-2206 for 48 hours. Scale bar: 145 µm. **b** Viability (% of control) of 4T1-Luc2 cells treated as described in (a) (n = 3). Means ± SEM. *P < 0.05, **P < 0.01. (b: one-way ANOVA with Tukey’s multiple comparisons test).

**
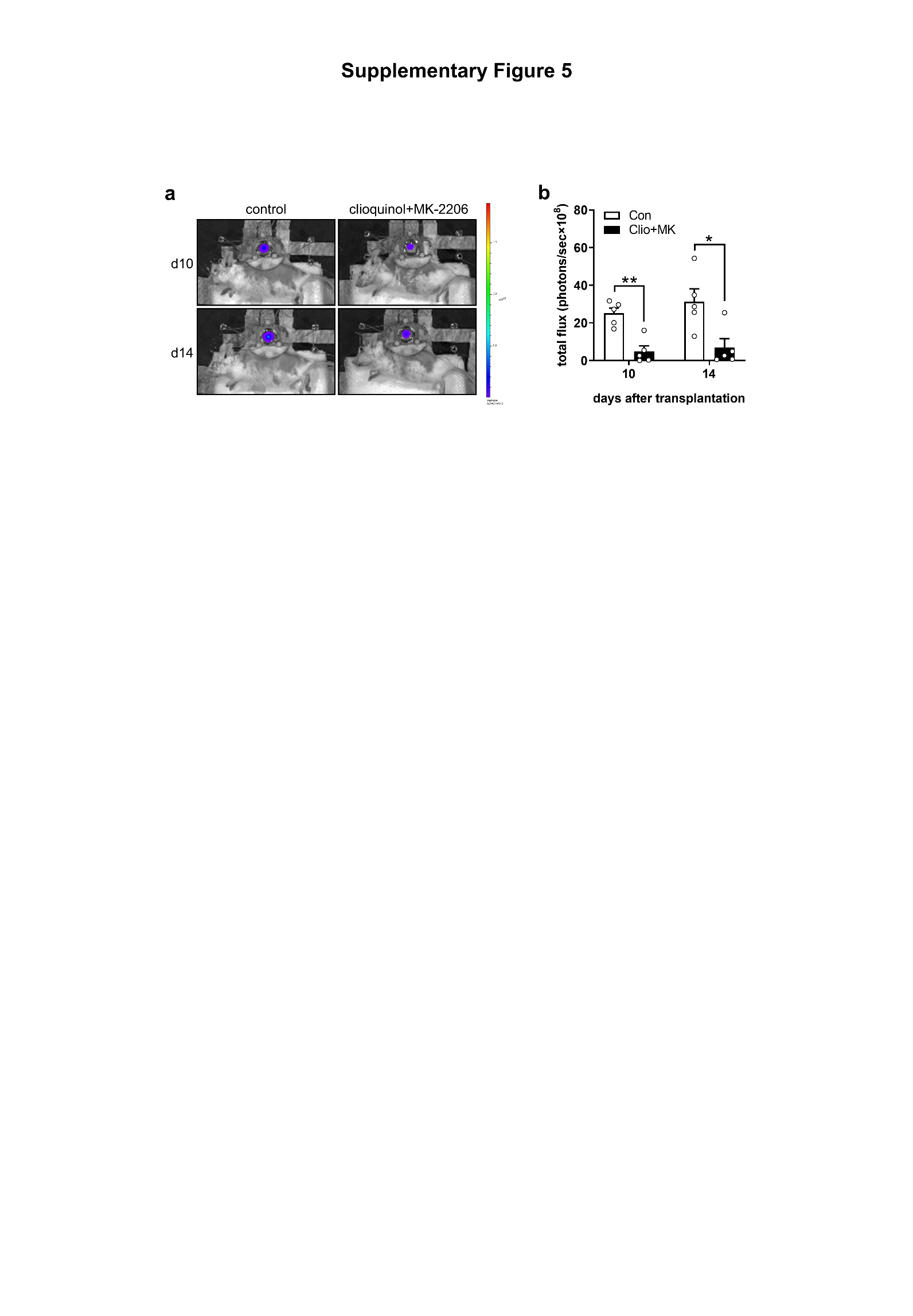
**

**Supplementary Fig. 5** Combined effects of clioquinol and MK-2206 on TNBC development, as assessed by bioluminescence imaging. **a** Bioluminescent images of 4T1 tumors in control and combination group on days 10 and 14 after tumor transplantation. **b** Total flux (photons/sec x 10^8^) over the tumor in control and combination groups, as described in (a) (n = 5). Means ± SEM. *P < 0.05, **P < 0.01. (b: unpaired Student’s t-test).
