# Supplementary material for: Clioquinol inhibits angiogenesis by promoting VEGFR2 degradation and synergizes with AKT inhibition to suppress triple-negative breast cancer vascularization
Source: Angiogenesis. 2025 Feb 3;28(2):13. doi: 10.1007/s10456-024-09965-1 (PMC11790708; doi:10.1007/s10456-024-09965-1)
Supplement: Supplementary file 2 — Supplementary Material 2 [file 10456_2024_9965_MOESM2_ESM.docx]

**Supplementary Table 1. Calculation of CDI values.**

| **figure** | **ratio of each group to the control group** | | | **CDI** |
| --- | --- | --- | --- | --- |
|  | clioquinol | MK-2206 | clioquinol + MK-2206 |  |
| Fig. 6d | 0.633 | 0.648 | 0.421 | 1.026 |
| Fig. 6f | 0.678 | 0.700 | 0.377 | 0.795 |
| Fig. 7b | 0.560 | 0.501 | 0.288 | 1.027 |
| Fig. 7d | 0.664 | 0.810 | 0.420 | 0.781 |
